# The *Agaricus bisporus cox1* Gene: The Longest Mitochondrial Gene and the Largest Reservoir of Mitochondrial Group I Introns

**DOI:** 10.1371/journal.pone.0014048

**Published:** 2010-11-18

**Authors:** Cyril Férandon, Serge Moukha, Philippe Callac, Jean-Pierre Benedetto, Michel Castroviejo, Gérard Barroso

**Affiliations:** 1 UMR 5234 CNRS (Centre National de la Recherche Scientifique) – Université Victor Segalen Bordeaux 2, Bordeaux, France; 2 Laboratoire de Toxicologie et Hygiène Appliquée, UFR des Sciences Pharmaceutiques, Université Victor Segalen Bordeaux 2, Bordeaux, France; 3 INRA (Institut National de la Recherche Agronomique) UR 1264 Mycologie et Sécurité des Aliments, Villenave d'Ornon, France; The J. Craig Venter Institute, United States of America

## Abstract

In eukaryotes, introns are located in nuclear and organelle genes from several kingdoms. Large introns (up to 5 kbp) are frequent in mitochondrial genomes of plant and fungi but scarce in Metazoa, even if these organisms are grouped with fungi among the Opisthokonts. Mitochondrial introns are classified in two groups (I and II) according to their RNA secondary structure involved in the intron self-splicing mechanism. Most of these mitochondrial group I introns carry a “Homing Endonuclease Gene” (*heg*) encoding a DNA endonuclease acting in transfer and site-specific integration (“homing”) and allowing intron spreading and gain after lateral transfer even between species from different kingdoms. Opposed to this gain mechanism, is another which implies that introns, which would have been abundant in the ancestral genes, would mainly evolve by loss. The importance of both mechanisms (loss and gain) is matter of debate. Here we report the sequence of the *cox1* gene of the button mushroom *Agaricus bisporus*, the most widely cultivated mushroom in the world. This gene is both the longest mitochondrial gene (29,902 nt) and the largest group I intron reservoir reported to date with 18 group I and 1 group II. An exhaustive analysis of the group I introns available in *cox1* genes shows that they are mobile genetic elements whose numerous events of loss and gain by lateral transfer combine to explain their wide and patchy distribution extending over several kingdoms. An overview of intron distribution, together with the high frequency of eroded *heg*, suggests that they are evolving towards loss. In this landscape of eroded and lost intron sequences, the *A. bisporus cox1* gene exhibits a peculiar dynamics of intron keeping and catching, leading to the largest collection of mitochondrial group I introns reported to date in a Eukaryote.

## Introduction

Introns have been described in prokaryotes, eukaryotes and even in viral genomes [1], [2], [3], [4]. In eukaryotes, they are present in nuclear, plastidial and mitochondrial genes from several kingdoms and with various sizes and frequencies [5]. Large introns (from 0.1 to 5 kbp) are frequently found in mitochondrial genes of plant and fungal kingdoms [5]. However, they are scarce in the mitochondrial genomes of Metazoa, despite being grouped with fungi among the Fungi/Metazoa (Opisthokonts) super-kingdom. Mitochondrial introns are classified in two main groups (group I and II) according to their RNA secondary structure involved in the intron self-splicing mechanism [5], [6]. Mitochondrial group I and group II introns can be found in the same species and even in the same gene. Fungal mitochondrial genes tend to bear group I introns while plant mitochondrial genes tend to bear group II introns [5]. For example, the *cox1* gene of the fungus *Podospora anserina* possesses 15 group I introns and one group II [7] and the complete mitochondrial genome of the liverwort *Marchantia polymorpha* carries 25 group II and seven group I [8]. Most of the mitochondrial group I introns carry a “Homing Endonuclease Gene” (*heg*) encoding a DNA endonuclease (HE) acting in the transfer and site-specific integration (“homing”) of the intron [9], [10], [11]. The presence of these *heg* allowed the raising of a theory where introns might be mobile elements able to spread and to be gained by genes after lateral transfer even between phylogenetically distant species from different kingdoms [12], [13]. However, this theory does not infer the origin of the mobile intron. Another theory suggests that introns were abundant in the ancestral genes and mainly evolve by loss [14], [15]. However, both hypotheses are matters of much debate about the dynamics of intron loss and gain during evolution [16], [17]. For example, two recent studies dealing with a group I intron widely distributed in plant angiosperms led to conflicting conclusions [16], [17]. Indeed, the history of these sequences is thought to be dominated by numerous events of loss [16] or due to frequent gains after a lateral transfer from a fungal donor [17].

The *cox1* gene (encoding the subunit 1 of the cytochrome c oxydase) was widely used in a successful “DNA barcoding” taxonomic method in animals [18]. However, in the related fungal kingdom, this approach is hampered by the presence of several large group I introns [19]. Indeed, the fungal *cox1* gene is the mitochondrial gene showing the highest number of introns [19]. Owing to this wealth of introns, few fungal complete *cox1* gene sequences are available in databases, especially for species belonging to the Basidiomycota division where only nine sequences (*Cryptococcus neoformans* var. *neoformans*, *Cryptococcus neoformans var. grubii*, *Ustilago maydis*, *Tilletia indica*, *Moniliophtora perniciosa*, *Pleurotus ostreatus*, *Agrocybe aegerita*, *Schizophyllum commune* and *Trametes cingulata*) have been reported and correctly annotated to date.

To understand the evolution and dynamics of mitochondrial introns in fungi, we decided to determine the sequence and molecular organization of the commonly used *cox1* model gene in the most widely cultivated mushroom in the world: *Agaricus bisporus*.

## Results and Discussion

### The *A. bisporus cox1* gene: the largest mitochondrial intron reservoir

**Figure 1:**
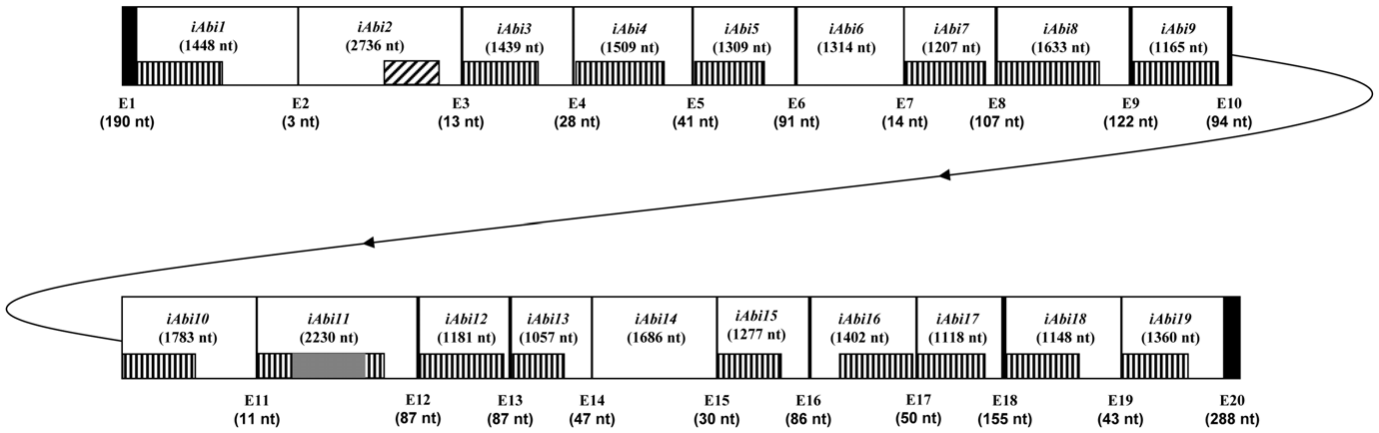
Molecular organization of the *Abi cox1* gene. Vertical lines represent exons and their nucleotide (nt) sizes are indicated below the exon E1 to E20. The name and nt size of the introns are indicated in the empty boxes. The putative functional *heg* in group I introns are shown by vertical hachured boxes, and the eroded reverse transcriptase gene carried by the *iAbi2* group II intron by a diagonal hachured box. The invading *heg* with a *cob* origin harbored by the *iAbi11* intron is shown by a grey box.

The complete sequence (29.902 nt, GenBank Accession number: EU314927) of the *cox1* gene of the button mushroom *Agaricus bisporus (Abi)* was determined for a subculture (Bs 518) of the traditional brown cultivar C9 from overlapping cloned *Bam*HI and *Eco*RI restriction fragments and cloned PCR products (Supplementary information file, Figure S1). An alignment of this gene with the *cox1* exon sequence (CDS) of the related Agaricales *Agrocybe aegerita* [12] shows that the *Abi cox1* CDS is split by 19 large (>1 kbp) introns. The borders of these introns, their group and sub-group were deduced from the design of each secondary structure (Supplementary information file, Figure S2) and from a comparison of the gene with the *Abi* cDNA (1,587 nt). The 19 introns are scattered over the whole gene sequence (Figure 1). The size of the exons varies from 3 nt (exon 2) to 288 nt (exon 20), contrasting with the large size of the introns that ranges from 1,057 nt (*iAbi*13) to 2,736 nt (*iAbi*2). The intron sequences represent 28,318 nt, i.e. 94.7% of the gene. The *Abi cox1* gene is the longest mitochondrial gene reported to date in all kingdoms. Indeed, the next longest mitochondrial genes are the *cox1* gene of the Ascomycota *Podospora anserina* (24,507 nt) which carries 14 group I and one group II introns [7] and the Basidiomycota *Trametes cingulata* (22,370 nt) with 15 group I introns [20].

### Intact versus eroded intron encoded Open Reading Frames (ORFs)

Only one (*iAbi2)* of the 19 introns belongs to group II (subgroup IIA1). This large intron (2,736 nt) contains an eroded ORF (402 aa) encoding a putative protein possessing significant amino acid (aa) sequence identity (up to 38%) with the central and C-terminal regions of several reverse transcriptases (RT) encoded by plant and fungal mitochondrial group II introns. However, this encoded protein lacks the first 200 aa of the RT N-terminal region. All these RT genes are carried by group II introns possessing the same insertion site in the orthologous *cox1* genes. The highest amino acid (aa) identities (id.) and similarities (sim.) of the *iAbi2* putatively encoded RT were obtained with the phylogenetically distant Ascomycota *Cryphonectria parasitica* (38% aa id., 56% aa sim.) and the liverwort (Viridiplantae) *Marchantia polymorpha* (37% aa id., 54% aa sim.). In *Abi*, this intron might be undergoing an elimination process, as suggested by the erosion of the encoded reverse transcriptase.

Fifteen out of the 18 group I introns of the *Abi cox1* gene harbor intact ORF encoding a putative functional HE, while only three (*iAbi6*, *iAbi11* and *iAbi14)* contain eroded *heg* leading to non-functional HE (Figure 1). The eroded *heg* are characterized by the presence of several length and point mutations leading to frameshifts and to the appearance of stop codons interrupting the ORF.

### Evidence for recent invasion of an *A. bisporus cox1* intron by a *heg* arising from another gene

The *heg* encoded by the *iAbi11* intron exhibits a peculiar organization, resulting from the insertion in a *cox1 heg* of an other invading *heg* (Figure 1). This intron ORF of 1,800 nt (nt 16,249–18,478) is constituted by the insertion (at nt 16,848) in the *iAbi11 cox1* intron *heg* (recipient) of a second complete *heg* (invader). Both *heg* are in an open reading frame.

The *iAbi11* recipient *heg* possesses a percentage of aa identity ranging from 73% with *heg* encoded by the Ascomycota *Podospora anserina i9* or *Gibberella zeae i5* to 40% for an orthologous group I intron widely distributed in plants. All these orthologous *heg* are carried by introns located at the same position in fungal and plant *cox1* genes.

The *iAbi11* invading *heg* is orthologous to a *heg* carried by an intron located in another mitochondrial gene: the *cob* gene of several Ascomycota species. The highest percentage of aa identity is obtained with *G. zeae* (50%) and *P. anserina* (47%).

In these two species, the invading and recipient *heg* are located in introns of the *cob* and *cox1* genes, respectively. On the contrary, in *A. bisporus*, both heg are located in the same intron of the *cox1* gene.

As the recipient and invading *heg* do not possess sequence homology, the *iAbi11* chimeric *heg* can not be generated by a recombinational event. Hence, the invasion of the *iAbi11* intron *heg* by a *heg* arising from an other gene (*cob*) suggests a recent transfer activity of the enzyme encoded by the mobile invading element, as previously reported in the T4 phage and in the fungus *Podospora curvicolla* [21], [22].

### Landscape of eukaryotic mitochondrial group I introns: a patchy distribution

**Figure 2:**
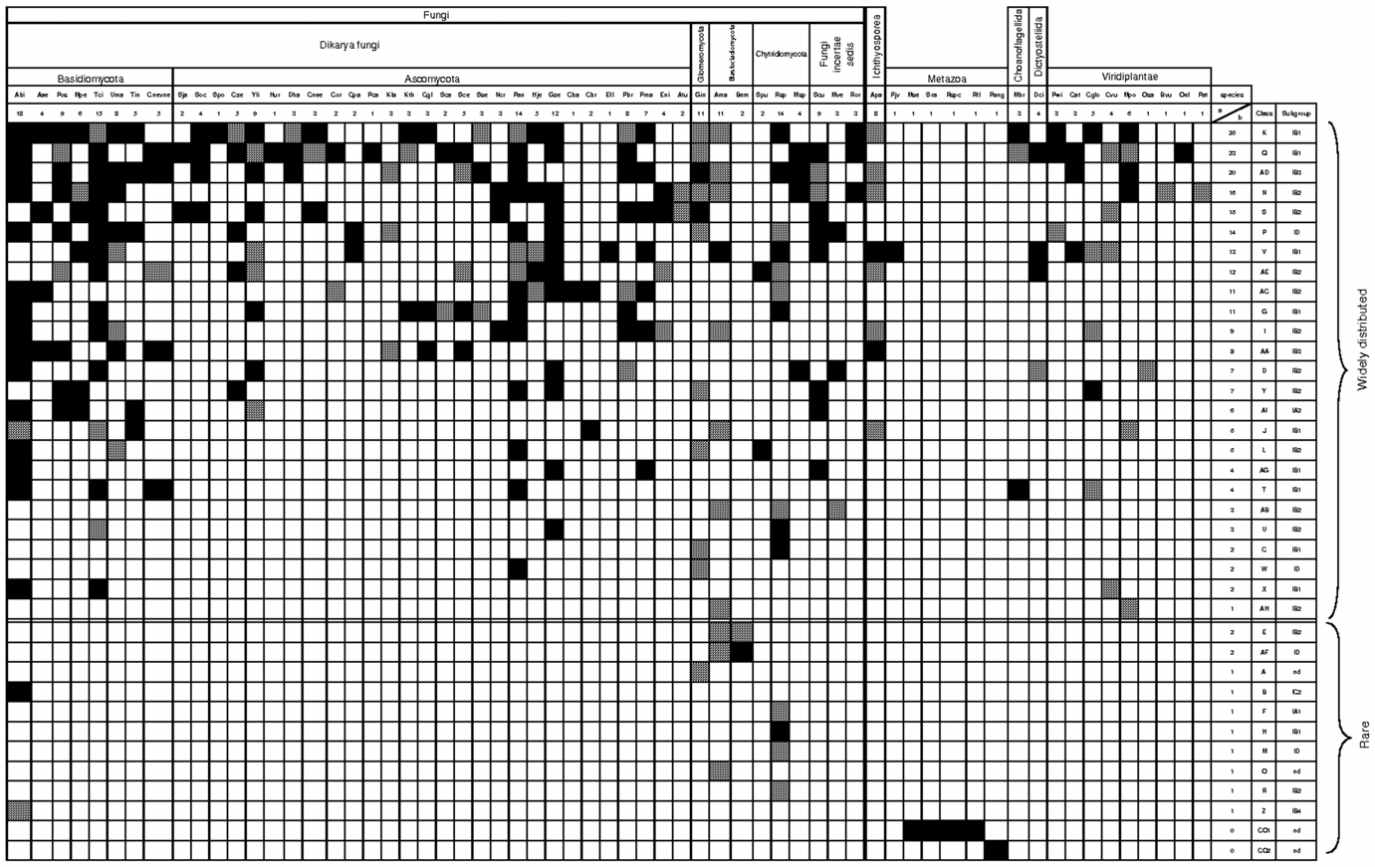
Schematic representation of the distribution of group I introns, according to their Pcl (row) and to the species harboring them (column). Row “a” indicates the number of introns per species while column “b” shows the number of fungal introns per Pcl. All introns of the same Pcl are in the same row and all introns carried by a given species are in the same column. The first 35 Pcls were named by increasing alphabetic characters from the 5′-end of the *cox1* CDS. The 37 rows of the Pcls are organized according to the decreasing number of fungal representatives they contain from the most distributed Pcl K (25 members) to the 12 Pcls of “rare” introns containing either a single member or two (E and AF Pcls) to four members (in both additional Pcls CO1 and CO2) shared by closely related species. Presence of intact and eroded/or absent *heg* are shown by black and hachured boxes, respectively. Species were organized according to their taxonomic (superkingdoms and kingdoms, phylla, orders) and their phylogenetic positions, as indicated in Figure 4 A and B. Fungi Dikarya Basidiomycota: *Agaricus bisporus* (EU314927), Aae: *Agrocybe aegerita* (AF010257), Pos: *Pleurotus ostreatus* (NC_009905), Mpe: *Moniliophthora perniciosa* (NC_005927), Tci*: Trametes cingulata* (NC_013933), Uma: *Ustilago maydis* (NC_008368), Tin: *Tilletia indica* (NC_009880), Cnevne: *Cryptococcus neoformans* var *neoformans* (AY560609). Ascomycota: Sja: *Schizosaccharomyces japonicum* (NC_004332), Soc: *Schizosaccharomyces octosporus* (NC_004312), Spo: *Schizosaccharomyces pombe* (NC_001326), Cze: *Candida zemplinina* (NC_005972), Yli: *Yarrowia lipolytica* (NC_002659), Hur: *Hanseniaspora urvum* (NC_007780), Dha: *Debaryomyces hansenii* (NC_010166), Cnee: *Candida neerlandica* (YP_002122387), Cor: *Candida orthopsilosis* (DQ026513), Cpa: *Candida parapsilosis* (NC_005253), Pca: *Pichia canadensis* (NC_001762), Kla: *Kluyveromyces lactis* (NC_006077), Kth: *Kluyveromyces thermotholerans* (NC_006626), Cgl: *Candida glabrata* (NC_004691), Sca: *Saccharomyces castellii* (NC_003920), Sce: *Saccharomyces cerevisiae* (NC_001224), Sse: *Saccharomyces servazii* (NC_004918), Ncr: *Neurospora crassa* (X14669), Pan: *Podospora anserina* (NC_001329), Hje: *Hypocrea jecorina* (NC_003388), Gze: *Gibberella zeae* (NC_009493), Cba: *Cordyceps bassiana* (ABU50156), Cbr: *Cordyceps brongniartii* (YP_002213602), Efl: *Epidermophyton floccosum* (NC_007394), Pbr: *Paracoccioides brasiliensis* (NC_007935), Pma: *Penicillium marneffei* (NC_005256), Eni: *Emericella nidulans* (X00790), Atu: *Aspergillus tubingensis* (NC_007597). Glomeromycota: Gin: *Glomus intraradices* (NC_012056). Blastocladiomycota: Ama: *Allomyces macrogynus* (NC_001715), Bem: *Blastocladiella emersonii* (YP_002274319). Chytridiomycota: Spu: *Spizellomyces punctatus* (NC_003052), Rsp: *Rhizophydium sp*. 136 (NC_003053), Msp: *Monoblepharella sp.* (NC_004624). Fungi incertae sedis: Scu: *Smittium culisetae* (NC_006837), Mve: *Mortierella verticillata* (NC_006838), Ror: *Rhizopus oryzae* (NC_006836). Ichthyosporea: Apa: *Amoebidium parasiticum* (AAN04062). Eumetazoa: Pjv: *Palythoa sp*. JVK-2006 (ABF67639). Corals: Mse: *Metridium senile* (NP_009253), Sra: *Siderastrea radians* (YP_654418), Rspc: *Rhodactis sp*. CASIZ 171755 (YP_654290), Rfl: *Ricordea florida* (YP_654303), Pang: *Plakortis angulospiculatus* (YP_001648679). Choanoflagellida: Mbr: *Monosiga brevicollis* (NP_696984). Dictyosteliida: Dci: *Dictyostelium citrinum* (NC_007787). Viridiplantae: Pwi:*Prototheca wickeramii* (NP_042244), Cat: *Chlorokybus atmophyticus* (YP_001315139), Cglo: *Chaetosphaeridium globosum* (NP_689386), Cvu: *Chara vulgaris* (NP_943703), Mpo: *Marchantia polymorpha* (NC_001660), Osat: *Oryza sativa* (BAD38494), Bvu: *Beta vulgaris* (NP_064063), Oel: *Ochrosia elliptica* (ABY83864), Patr: *Plantago atrata* (ABY83853). The following fungal species have intronless *cox1* genes: *Schizophyllum commune* (NC_003049), *Cryptococcus neoformans* var *grubii* (NC_004336), *Ashbya gossypii* (NC_005789), *Beauveria bassiana* (YP_001876504), *Mycosphaerella graminicola* (NC_010222), *Hyaloraphidium curvatum* (NP_150103), *Harpochytrium sp*. JEL94 (NC_004760), *Harpochytrium sp*. JEL105 (NC_004623).

To understand the origin and dynamics of the *Abi* group I introns, all the complete *cox1* genes in databases were compiled (Figure 2) as described in the Materials and Methods section. This showed that the mitochondrial group I introns have been reported in three kingdoms: Fungi/Metazoa, Viridiplantae and Amoebozoa. In the Fungi/Metazoa, mitochondrial group I introns have been described in a single species of the Choanoflagellida order (*Monosiga brevicollis*) [23], in one species of the Porifera phylum (Metazoa kingdom) [24] and in numerous species of the fungal kingdom [19]. In contrast, all the *cox1* genes from the Eumetazoa lack group I intron, except two different intron types described in Cnidaria [25], [26], [27].

240 group I introns were found in 53 fungal *cox1* genes (including three species of the Zygomycota phylum, now fungi incertae sedis). They were sorted in 35 position classes (Pcls) defined first by the same location in the *cox1* CDS sequence. This distribution, which is based on the precise insertion site of each intron, is correlated with the highest sequence identities at the nucleotide level as well as with the amino acid identities of the putative encoded HE. Hence, each Pcl is also a sequence identity class. Indeed, each Pcl was shown to be constituted by introns inserted at the same position in the *cox1* CDS and, when present, carrying an orthologous *he*g. The orthologous character was deduced first from BLASTx analysis. When using a *heg* described in a *cox1* intron as a query in a BLASTx search, the homologous sequences with the higher scores (always higher than 80) and consequently, the lowest E-values (<e-20) were analyzed. In the large majority of cases, these sequences corresponded to *heg* located in introns with the same location in the *cox1* CDS. If the sequence of the *cox1* gene was complete and fully annotated, these *heg* were included in the same Pcl. Moreover, the analysis of all these complete *cox1* genes allowed the highlight of some additional *heg* whose encoded protein was eroded or undescribed. The corresponding introns were added to the corresponding Pcl. In a few cases, a sequence with similar E-values but located in a mitochondrial gene different from the *cox1* gene (*nad5* or *cob*) was present in the BLAStx results. These sequences have been considered as an invader with a *cox1* intron origin and not included in our analysis of the *cox1* introns. In all cases, we did not find any non-homologous intron *heg* located at the same precise site of the *cox1* CDS. This is in accordance with the fact that the insertion site of the intron appears to be determined by the DNA sequence precisely recognized by its encoded HE [2], [28]. As expected, the BLASTx results were confirmed by ClustalW alignments of the HE showing that the percentages of aa identity of all the intact HE belonging to a Pcl were always higher than those obtained when aligned with HE from the other Pcls (Supplementary information file, Figure S3).

**Figure 3:**
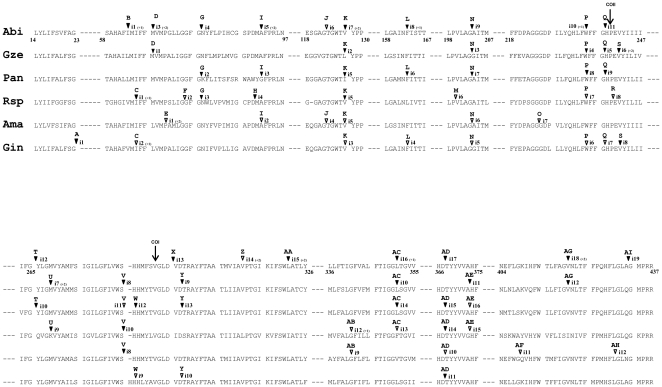
Comparison of group I intron insertion sites in the amino acid alignment (Clustal W) of the COX1 protein encoded by six long *cox1* genes of fungi. Introns are designated by their Pcl name (From A to Z and from AA to AI) and by their number in each fungal gene starting from the 5′ end of the gene. Closed and open symbols (triangles) indicate introns with intact and eroded *heg*, respectively. The insertion sites of the coral introns Pcls CO1 and CO2 never reported to date in the fungal kingdom are indicated by an arrow. The symbols “+1” and “+2” indicate that the insertion of the intron occurs inside the indicated codon: between the nt 1 and nt 2 of this codon for “+1” and between the nt 2 and nt 3 for “+2”.

The 35 Pcls were named in alphabetic order starting from the 5′-end of the *cox1* CDS. Since all the 35 Pcls are represented in at least one of the six longest fungal *cox1* genes, an alignment of the corresponding COXI proteins is used in Figure 3 to represent their relative positions. The six longest *cox1* genes are found in the Basidiomycota *Abi* (18 group I introns), in the Ascomycota *Podospora anserina* (*Pan*: 14 introns) and *Gibberella zeae* (*Gze*: 12 introns), in the Chytridiomycota *Rhyzophidium sp.* (*Rsp*: 14 introns), in the Blastocladiomycota *Allomyces macrogynus* (*Ama*: 11 introns) and in the Glomeromycota *Glomus intraradices* (*Gin*: 11 introns).

The number of fungal *cox1* introns assigned to each Pcl varied greatly from 25 in the Pcl K to a single one in 8 different Pcls (Figure 2). Hence, these introns can be classified as being either rare or widely distributed. Rare introns are those detected either in a single species (8 Pcls) or in two closely related species for the Pcls E and AF, which both contain introns reported in two Blastocladiales. The introns of the remaining 25 Pcls show a wide and patchy distribution ranging from 2 in the Pcl W to 25 in the Pcl K, and they are present in different phyla of the fungal kingdom. At the same time, phylogenetically close species differ by the presence/absence of these introns. For instance, the two representatives of Pcl W have been described in the Glomeromycota *Gin* and in the distant Ascomycota *Pan* (Figure 2). The 25 representatives of the largest Pcl K are present in one species (*Rsp*) out of six Chytridiomycota, in one species out of two Blastocladiella, in the Glomeromycota species, in one out of three species of the basal fungal lineages, in seven out of ten Basidiomycota and in 14 out of 31 Ascomycota. *Abi* contains15 introns belonging to the 25 widely distributed Pcls, while *Schizophyllum commune* (*Sco*) which belongs to the same Agaricales order is intronless. Thus, the observed wide and patchy distribution of the group I fungal introns appears to result from numerous losses and gains of these mobile genetic elements.

Among the 35 fungal intron Pcls, 17 contained orthologous introns defined by the same position in the *cox1* CDS in the Viridiplantae, Amoebozoa or Metazoa kingdoms. As previously established with fungal introns (i.e. deduced from BLASTx results and confirmed by percentages of aa identities of the encoded HE that were always higher inside a Pcl than between Pcls), the *heg* of the introns from all these kingdoms belonging to the same Pcl are orthologous genes. These Pcls represent 49% (17/35) of the Pcls and up to 60% (15/25) of the widely distributed ones. Except for two group I introns specifically harbored by coral species (classes Co1 and Co2), all the *cox1* group I introns belong to Pcls previously defined by representatives from the fungal kingdom.

**Figure 4:**
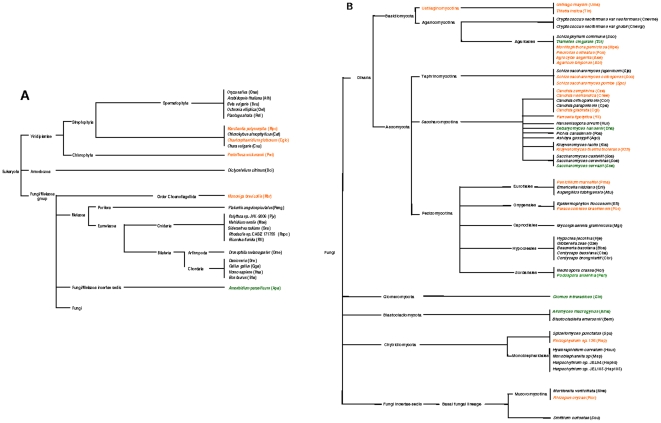
Taxonomic and phylogenetic position of species with and without a Pcl K intron. In Figure 4A and 4B, the species harboring a Pcl K orthologous intron with an intact or an eroded *heg* are in orange and green, respectively. The species lacking an orthologous intron are in black. The phylogenetic position of each fungal species was established according to the phylogenetic tree reported by James et al. [29] for the fungal kingdom using a six-gene phylogeny. When not present in the tree, species were located according to the nearest taxon (same genus or order) found in the tree.

In Figure 2, the species were ordered according to their systematic position indicated in the taxonomic and phylogenetic trees in Figure 4A and B. The phylogenetic relationships of the species were deduced from the six-gene phylogeny established by James et al. [29]. The occurrence of the orthologous introns of the most widely distributed Pcl K is shown in the trees (Figure 4). This clearly shows that the patchy distribution of the species with and without orthologous introns requires a combination of multiple events of intron loss and gain.

A high sequence divergence can be seen between the *heg* sequences of orthologous introns of the same Pcl. The HE encoded by the introns of Pcl K show percentages of aa identity (and similarity) ranging from 31% aa id. (54% aa sim.) between the fungal Ascomycota *Gze* and the Viridiplantae *Mpo* to 56% aa id (73% aa sim.) between the Basidiomycota *Abi* and *Aae*. These sequence divergences between orthologous introns of the same Pcl, which are distributed over up to four kingdoms but are also observed between closely related species, can be explained by (i) a high divergence of vertically inherited introns maintained in the compared species during the course of evolution or (ii) independent gains due to lateral transfer.

Moreover, most of the Pcls of the widely distributed introns (Figure 2) contain introns with a potentially functional *heg* and others with an eroded *heg*. The Pcl AG is an exception containing four representatives with only intact *heg*. Erosion of the *heg* is thought to be a preliminary step before the complete elimination of the intron [14]. For each Pcl, the presence of intact and eroded *heg*, even in closely related species strengthens the hypothesis that numerous events of loss and gain occurred during evolution.

Most *cox1* genes of the Viridiplantae, Amoebozoa and Metazoa kingdoms lack intron and only a small number of species harbor 1 to 6 group I introns. On the contrary, the majority (45 genes) of the 53 *cox1* sequences of the fungal kingdom possess one to 18 introns, while only eight genes do not contain any intron.

### Dynamics of intron mobility

**Figure 5:**
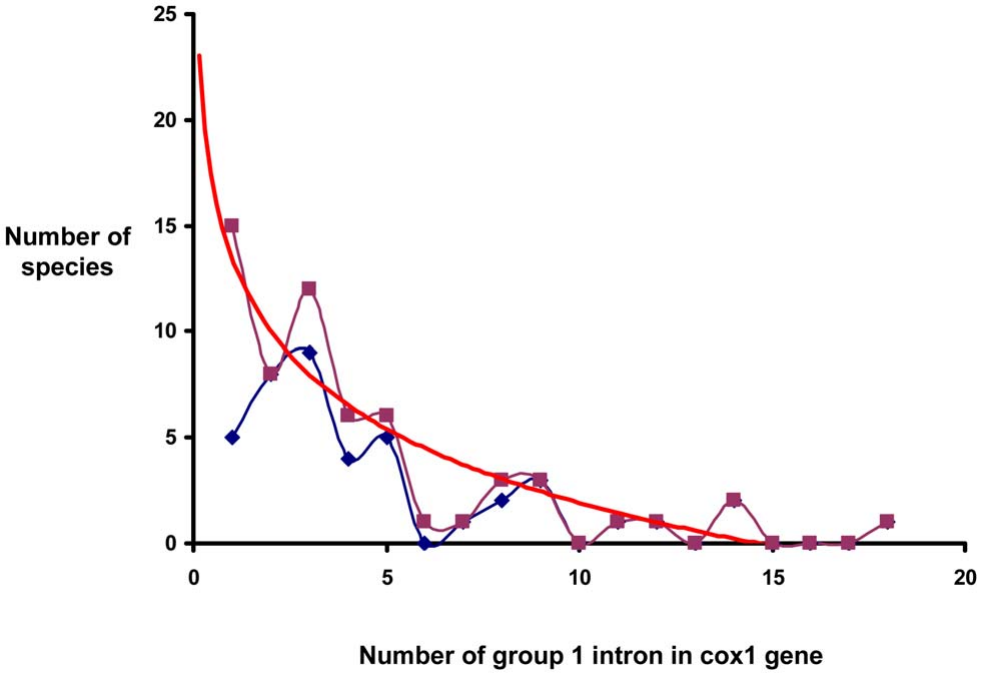
Graphical representation of number of eukaryotic species (purple curve) and fungal species (blue curve) as a function of number of introns carried by their *cox1* gene. The red curve shows the logarithmic regression model (y = −5,0316Ln(x) +13,507; R^2^ = 0.83) with all the eukaryotic species analyzed.

To analyse the dynamics (loss and gain) of group I introns in eukaryotes, a curve (Figure 5) representing the number of species according to the number of introns of their *cox1* gene was established. This curve does not fit with the Gaussian distribution that would be expected if the numbers of introns were to reflect random events of losses and gains. The plots can be represented by a theoretical curve showing a logarithmic decrease. Together with the high frequency of eroded *heg* carried by these introns (68 eroded *heg* among the 240 fungal introns analyzed), the curve can be interpreted as a trend of these group I introns towards loss from the *cox1* genes. However, the six long genes distributed over five different fungal divisions behave as group I intron reservoirs, with 11 to 18 introns. They contain at least one representative of each Pcl, except for the introns reported in corals. As the eight rare introns detected once were reported in five of these six long genes, the ability to bear specific or rare introns appears to be a characteristic of these reservoir species.

Moreover, most of the introns described in the long *cox1* genes from the basal fungal lineages carry eroded *heg*: 10 introns out of the 11 of *Ama*, 9 out of the 11 of *Gin* and 7 out of the 14 of *Rsp.* This suggests that these introns are being eliminated from these species. In principle, the erosion of the *heg* should definitively prevent the harboring intron from spreading. On the contrary, most of the introns of the long *cox1* genes of Dikarya (16/18, 12/14 and 12/12 for *Abi*, *Pan* and *Gze*, respectively) maintain a putative functional *heg*.

In this context, the *Abi cox1* gene has to be considered as the largest group I intron reservoir described to date. It possesses representatives of more than half (18/35 = 51%) of the Pcls, and more than 64% (16/25) of the widely distributed ones and all but two of its 18 group I introns possess intact and potentially functional *heg*.

### Conclusion

It appears that the evolution of the mitochondrial genome of eukaryotes is still moving in the direction of group I intron loss. However, in some species and particularly in fungi, complete elimination of the group I intron seems counterbalanced by the gain mechanism resulting from the lateral transfer of these mobile genetic elements. Furthermore, the long genes described in the Dikarya fungi, whose the *Abi cox1* gene is the most amazing representative to date, pose an appealing and still unresolved question about why and how some fungal species organize their mitochondrial genes in such an expensive manner.

## Materials and Methods

### DNA manipulation, molecular cloning and sequencing

The *Abi cox1* complete gene was sequenced by conventional procedures [30] from cloned mitochondrial restriction fragments and from PCR products as indicated in Supplementary information files Figure S1. All new data sequencing (i.e the complete sequence of the *Abi cox1* gene) has been deposited in the GenBank (Accession number EU314927).

The *Agaricus bisporus* strain Bs518 was cultured in Roux vials containing complete CYM medium supplemented with 10% (v/v) compost extract [31]. Mitochondrial DNA was purified from 5 g of vegetative mycelium according to Fukumasa-Nakaï et al.[32]. *Bam*H1 and *Eco*RI mitochondrial DNA libraries were constructed in *E. coli* XL1-Blue [33] using pGEM-7Zf(+) from Promega, according to [30]. Both libraries were screened by hybridization on colonies with probes constituted first by an *Abi cox1* cDNA and by cloned PCR products (Supplementary information files Figure S1).

*Abi cox1* cDNA was obtained by RT-PCR from 1.5 µg of total RNAs extracted by the “Hot Phenol” procedure [34] using the Access RT-PCR kit from Promega and a set of specific primers (2.5 pmol) designed from the *A. aegerita cox1* gene sequence (GenBank Accession number AF010257).

PCR amplifications were carried out using the Go *Taq* polymerase from Promega Corp. and couples of primers (Supplementary information files Figure S1) deduced from the sequences and synthesized by MWG (Seraing, Germany). PCR were performed in a Programmable Thermal Cycler PTC 200 (MJ Research Inc., Watertown, Mass., USA). Each reaction contained 10 to 30 ng of fungal genomic DNA, 4 µM of both primers, 200 µM of each dNTP, 1 unit of *Taq* DNA polymerase, in a final volume of 50 µl of the appropriate buffer. Reactions were run for 40 cycles at 95°C for 1 min, then two degrees below the Tm of both oligonucleotides for 1 min, 72°C for 1 min, and one final cycle at 72°C for 5 min. The PCR products were cloned on PGEMT-easy vector from Promega Corp. (Madison, Wisc, USA).

The different probes were recovered after agarose gel electrophoresis by using the “Wizard SV gel Kit”, then labeled with 925 kBq of [α^32^P] dCTP (110 TBq/mmol) from Amersham Pharmacia Biotech., using the Promega Corp. “Random Primers DNA Labeling Kit”. The probes had a specific radioactivity higher than 10^8^ cpm/µg DNA.

Cloned PCR products and the isolated mitochondrial genomic fragments were sequenced by the primer walking methods using the Big Dye® Terminator Cycle Sequencing kit v 1.1 (Applied Biosystems). Sequencing was performed in the genotyping and sequencing facility at University Victor Segalen Bordeaux 2.

### Sequence analyses

The overlapping *cox1* sequences were compiled and analyzed with the DNA Strider 1.2 software (Commissariat à l'énergie atomique, Gif-sur-Yvette, France). Comparisons with sequences of the GenBank and EMBL databases were performed using the BLAST search algorithm [35].

Compilation of the complete *cox1* gene sequences was performed by compiling the results (orthologous sequences) given by a BLASTx analysis using first the *A. bisporus cox1* CDS as query. This analysis revealed most of the fungal *cox1* sequences available in the Genbank and also numerous *cox1* genes from plants. Second, all the intron *heg* carried by these sequences were used independently as a query for BLASTx analysis. This allowed the recovery of some additional split *cox1* sequences from plants and also from other distant species such as Palythoa sp. JVK-2006 (Porifera), and species belonging to the Ichthyosporea, Choanoflagellida and Dictyosteliida phylla, which have been added to our analysis. Finally, the coral (Cnidaria) *cox1* genes were added to the analysis because they contain the only introns reported to date in the Eumetazoa kingdom without homology with introns from other kingdoms. Alignments of nucleotide and protein sequences were carried out with Clustal W (1.8) software [36].

The intron secondary structures (Supplementary information files Figure S2) were deduced from the determination of the conserved sequences P, Q, R and S of the intron cores and of the base-paired regions P1–P9 with the help of RNA-Weasel software, and by direct comparison with the standard scheme for group I introns [37] and with the secondary structures of the database established by Damberger and Gutell.[38].

## Supporting Information

Figure S1 Restriction map of the Abi mitochondrial genome region carrying the cox1 gene. The exon sequences are represented by coloured boxes. The cloned BamH1 and EcoRI restriction fragments used to determine the Abi cox1 gene sequence are indicated and named according to the restriction map. The sequenced PCR products and the location of the corresponding primers are indicated (CoxU: ATGAATTGGTTAAATTCTAC; 5'R: TTAAAAATGTAAACTCCTG, CoxR: TTATTAAGATTGCGCAGGT, B17U: TTAGTAGGATCCTCAGAG, E21R: AATATAAAGTACCTAAGGC; B13U: CCTTAAGATTGTAGAGTAG; B6R: ACAACAGATTATTTCTGGC; B6U: AATAAACTAACCCTACCAG; B15R: CTCTTAATATGATAAAGGTG; E19U: ATAAGATACTTAAGTCCCC; E14R: ATAAACTTAGCTACAGCC).
(0.18 MB PPT)

Figure S2 Secondary structures of the A. bisporus introns iAbi1 to iAbi19. Exonic sequences and intronic sequences are in lower-case and upper-case letters, respectively. The base-paired regions P1 to P9 are shown on the secondary structures, according to the standard scheme for group I introns described by Michel and Westhof [37]. For the group II intron iAbi2, the structural domains are indicated by roman numerals.
(0.74 MB PPT)

Figure S3 Alignement (A) and representation of the Total Score (B) obtained when comparing the 21 intact HE of the most widely distributed Pcl K. The Total Scores higher than 200 are in red, those between 80 and 200 in pink. In C are reported the percentages of aa identity (a) and aa similarity (b) between I-AbiVII-P and the 20 others orthologous HE.
(0.07 MB DOC)
