# Supplementary figures and images for: The Agaricus bisporus cox1 Gene: The Longest Mitochondrial Gene and the Largest Reservoir of Mitochondrial Group I Introns
Source: PLoS One. 2010 Nov 18;5(11):e14048. doi: 10.1371/journal.pone.0014048 (PMC2987802; doi:10.1371/journal.pone.0014048)

## Slide 1
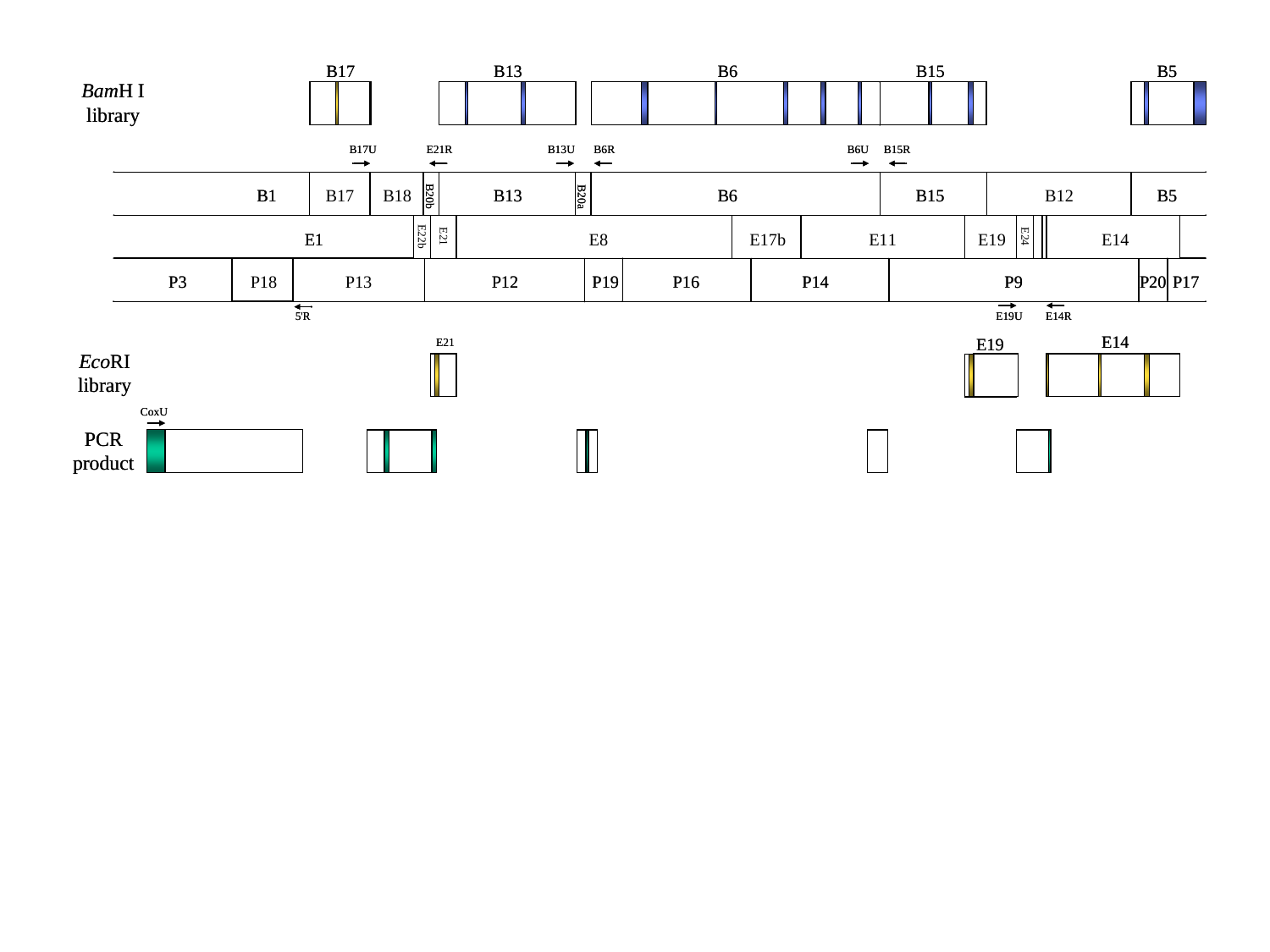

Supplement: Figure S1 — Restriction map of the Abi mitochondrial genome region carrying the cox1 gene. The exon sequences are represented by coloured boxes. The cloned BamH1 and EcoRI restriction fragments used to determine the Abi cox1 gene sequence are indicated and named according to the restriction map. The sequenced PCR products and the location of the corresponding primers are indicated (CoxU: ATGAATTGGTTAAATTCTAC; 5'R: TTAAAAATGTAAACTCCTG, CoxR: TTATTAAGATTGCGCAGGT, B17U: TTAGTAGGATCCTCAGAG, E21R: AATATAAAGTACCTAAGGC; B13U: CCTTAAGATTGTAGAGTAG; B6R: ACAACAGATTATTTCTGGC; B6U: AATAAACTAACCCTACCAG; B15R: CTCTTAATATGATAAAGGTG; E19U: ATAAGATACTTAAGTCCCC; E14R: ATAAACTTAGCTACAGCC). (0.18 MB PPT) [file pone.0014048.s001.ppt]
