# Supplementary material for: The Agaricus bisporus cox1 Gene: The Longest Mitochondrial Gene and the Largest Reservoir of Mitochondrial Group I Introns
Source: PLoS One. 2010 Nov 18;5(11):e14048. doi: 10.1371/journal.pone.0014048 (PMC2987802; doi:10.1371/journal.pone.0014048)
Supplement: Figure S2 — Secondary structures of the A. bisporus introns iAbi1 to iAbi19. Exonic sequences and intronic sequences are in lower-case and upper-case letters, respectively. The base-paired regions P1 to P9 are shown on the secondary structures, according to the standard scheme for group I introns described by Michel and Westhof [37]. For the group II intron iAbi2, the structural domains are indicated by roman numerals. (0.74 MB PPT) [file pone.0014048.s002.ppt]

## Slide 1
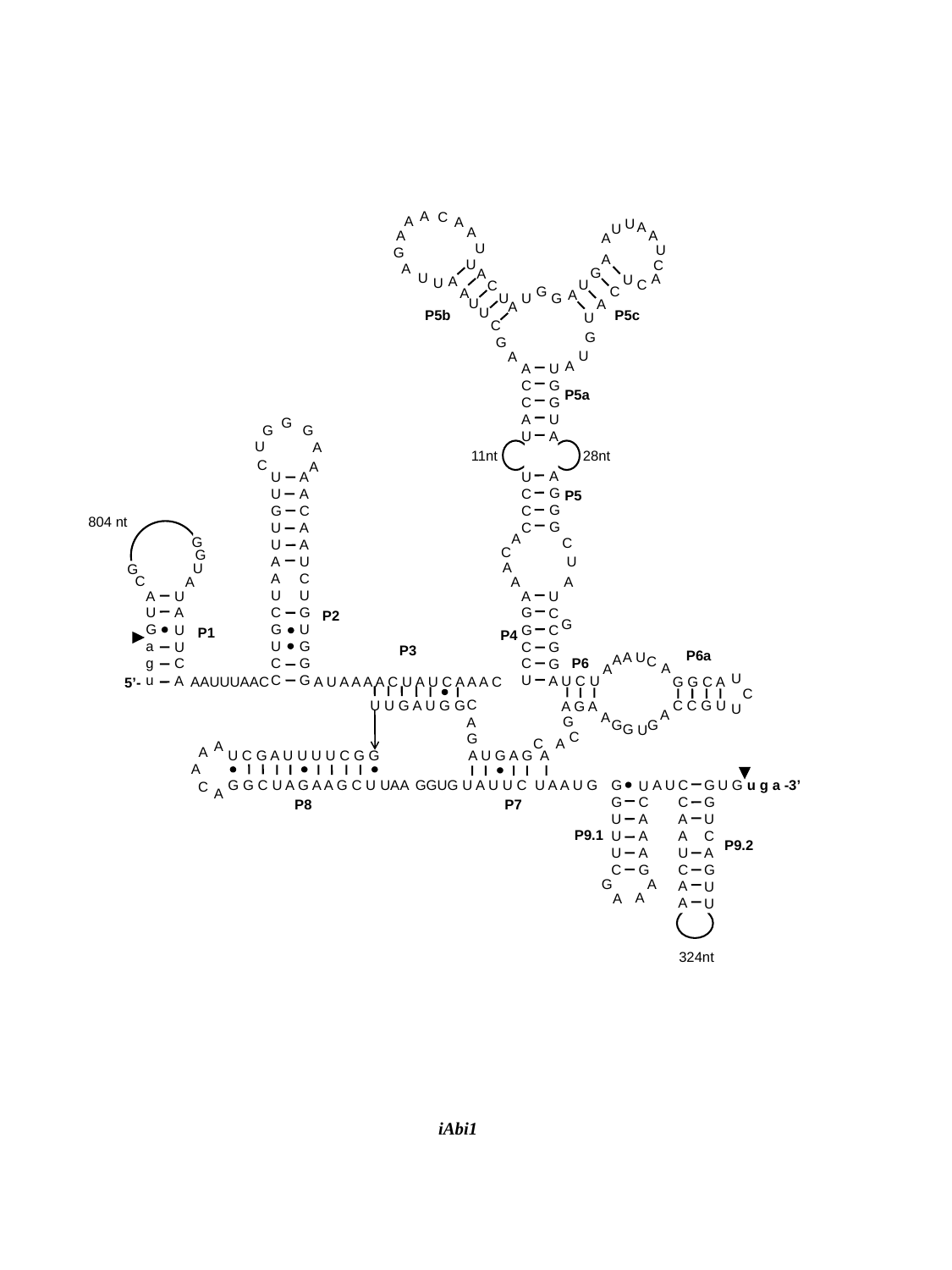

A
C
A
A
U
A
U
A
A
A
A
U
U
G
A
U
C
A
G
A
U
A
U
A
U
C
U
C
G
C
A
A
U
U
G
U
A
A
P5b
P5c
U
U
C
G
G
U
A
A
P5a
ACCAU
UGGUA
G
G
G
28nt
11nt
U
A
C
A
P5
804 nt
AGGG
UCCC
A
G
C
C
G
U
A
U
G
AACAAUCUGUGGG
A
UUGUUAAUCGUCC
C
A
A
P2
G
P1
P4
UAUUCA
AGGCCU
UCCGGA
AUGagu
P3
P6a
A
U
P6
A
C
A
A
U C U
A U A A A A C U A U C A A A C
G G C A
5’-
U
AAUUUAAC
C
U U G A U G G
C C G U
A G A
C
U
A
A
G
A
G
G
G
U
C
U G u g a -3’
G
C
A
A
U C G A U U U U C G G
A U G A G A
A
A
G G C U A G A A G C U UAA GGUG U A U U C U A A U G
A U
C
A
P8
P7
GGUUUC
UCAAAG
P9.1
CCAAUCAA
GGUCAGUU
P9.2
G
A
A
A
324nt
iAbi1

## Slide 2
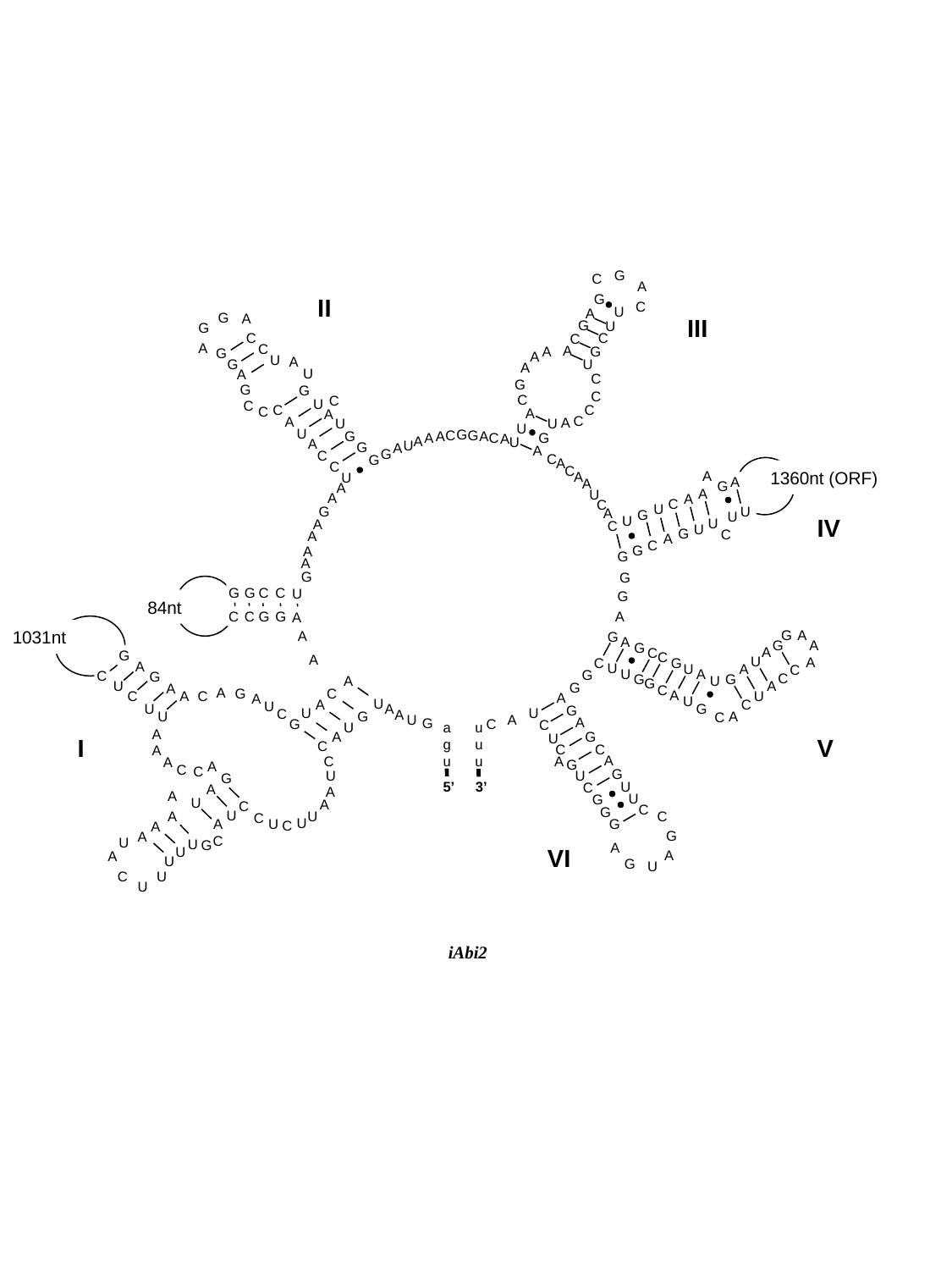

G
C
A
II
G
C
U
A
III
G
A
G
U
G
C
C
C
A
C
A
G
A
G
A
U
A
U
G
A
U
A
C
G
G
G
C
C
C
U
C
C
C
C
A
A
C
A
A
U
U
U
U
G
C
G
G
A
A
C
G
A
A
A
U
A
U
G
A
A
G
C
C
G
A
C
1360nt (ORF)
C
A
A
U
A
A
G
A
A
U
A
A
C
C
U
U
G
A
G
U
IV
U
U
A
C
U
G
C
A
A
C
G
A
G
A
G
G
G
G
C
C
U
G
84nt
C
C
G
G
A
A
1031nt
G
A
A
G
A
A
G
G
A
C
G
C
A
U
A
C
G
A
U
U
A
C
U
A
G
C
G
C
G
G
U
A
G
A
U
G
A
C
A
G
C
A
C
A
C
U
A
A
U
U
A
C
U
U
G
A
G
U
U
C
A
U
G
A
C
U
A
A
G
G
C
C
U
a
g
u
u
u
u
A
I
A
G
V
U
C
C
C
A
A
C
A
A
G
A
C
C
G
U
U
G
5’
3’
U
C
A
A
A
U
G
U
A
C
C
G
U
A
U
C
C
U
A
G
U
C
A
G
A
C
U
U
G
VI
A
U
A
A
U
G
U
C
U
U
iAbi2

## Slide 3
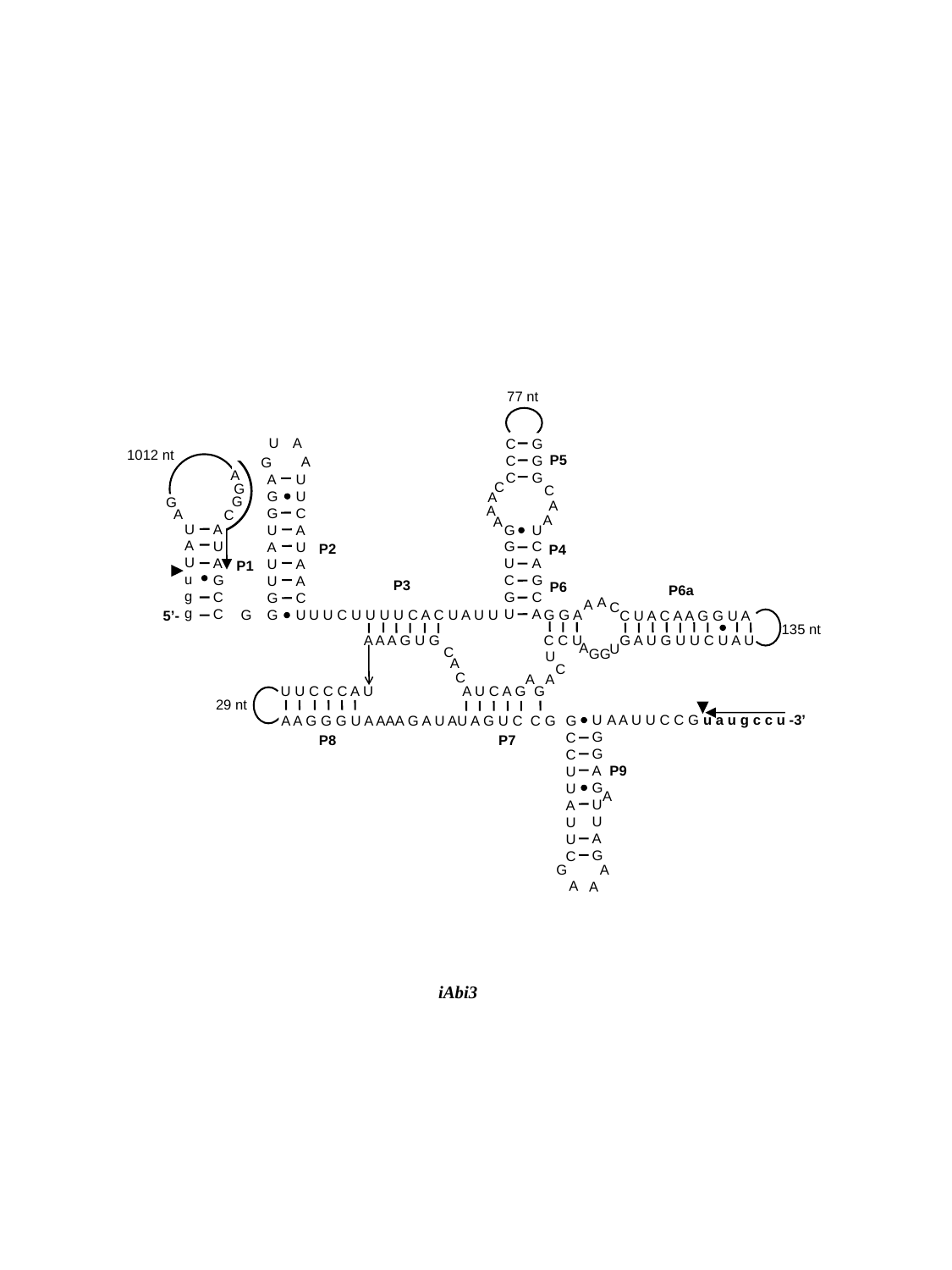

77 nt
1012 nt
A
U
P5
CCC
GGG
A
G
A
C
G
C
A
G
G
A
A
A
C
A
A
UUCAUAACU
P2
P4
AGGUAUUGG
P1
AUAGCC
GGUCGU
UCAGCA
UAUugg
P3
P6
P6a
A
A
C
135 nt
U U C U U U U C A C U A U U
G G A
5’-
C U A C A A G G U A
G
A A U U C C G u a u g c c u -3’
G A U G U U C U A U
A A A G U G
C C U
A
U
C
G
G
U
A
C
C
A
A
29 nt
A U C A G G
U U C C C A U
A AAA G A U AU A G U C C G
A A G G G U
P7
P8
P9
UGGAGUUAG
GCCUUAUUC
A
G
A
A
A
iAbi3

## Slide 4
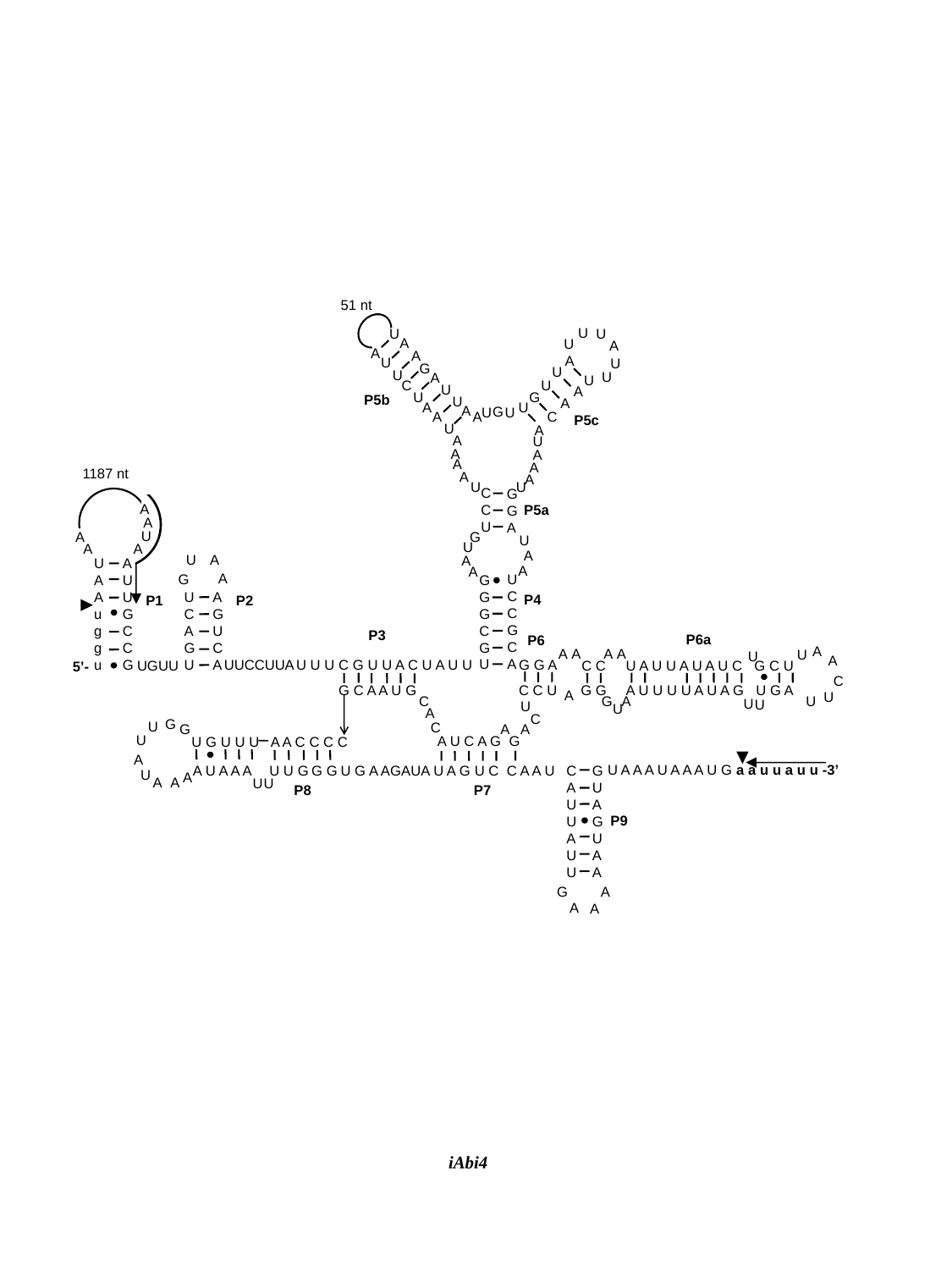

51 nt
U
U
U
A
U
A
A
A
A
U
U
G
U
U
U
A
U
C
U
U
A
P5b
U
G
U
A
A
U
A
G
U
U
P5c
C
A
A
U
A
A
U
1187 nt
A
A
A
A
A
A
U
U
CCU
P5a
GGA
A
A
U
A
G
U
U
A
A
A
A
U
A
A
A
A
G
P4
P2
P1
AUUGCCG
UAAuggu
UCCGCA
GGGCGU
AGUCA
P3
UCAGU
P6a
P6
U A A A U A A A U G a a u u a u u -3’
A
A
A
A
A
U
U
U U U C G U U A C U A U U
G G A
A
C C U A U U A U A U C G C U
5’-
UUCCUUA
UGUU
C
G G A U U U U A U A G U G A
G C A A U G
C C U
A
U
A
G
U
C
U
U
U
U
A
C
G
U
C
A
A
G
A U C A G G
U
U G U U U
A A C C C C
A
U G A AGAUA U A G U C C A A U
A U A A A
U U G G G
U
A
A
A
U
U
P7
P8
P9
CAUUAUU
GUAGUAA
G
A
A
A
iAbi4

## Slide 5
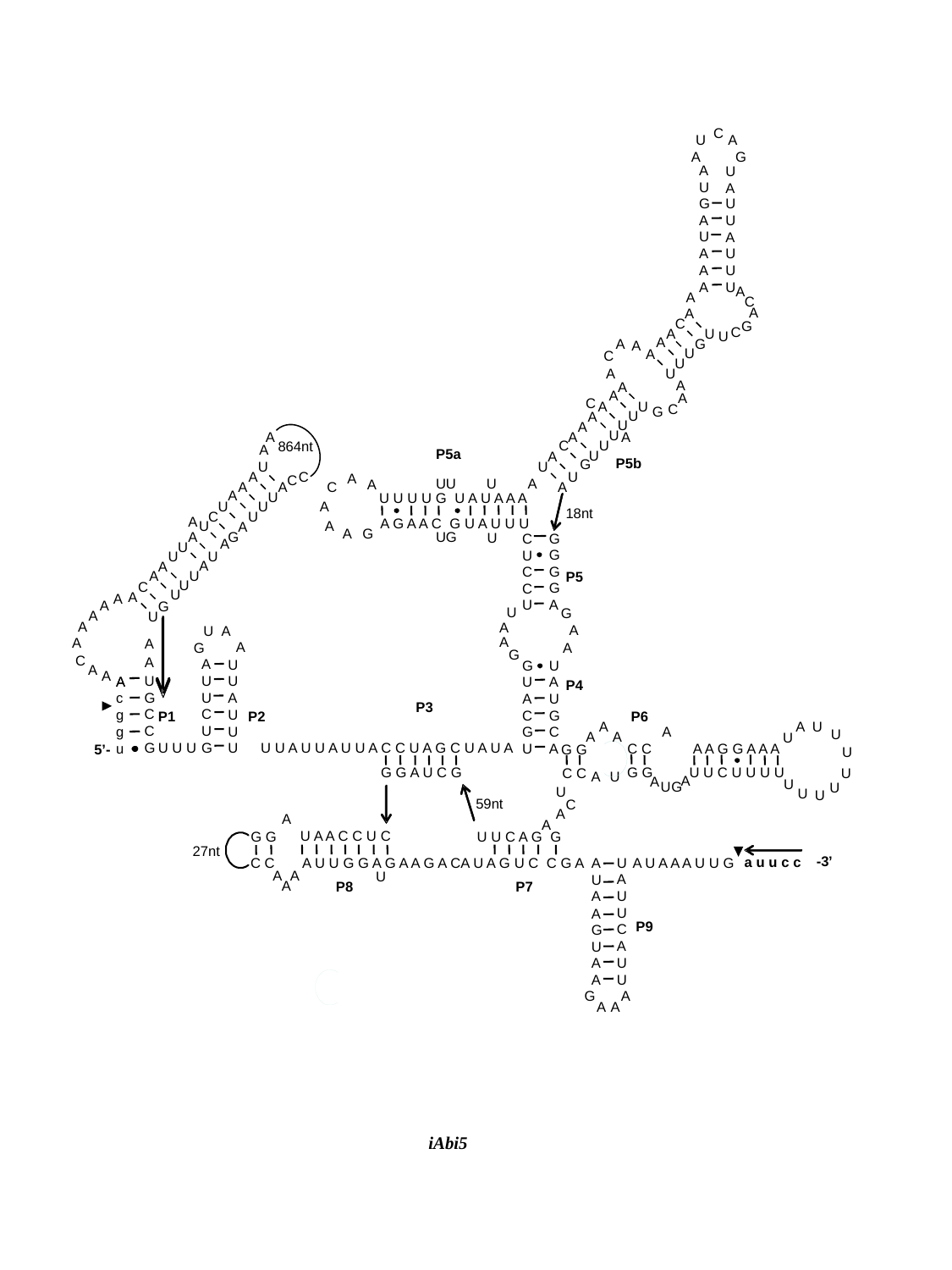

C
U
A
A
G
A
U
U
A
G
U
A
U
U
A
A
U
A
U
A
U
A
A
C
A
A
C
G
C
A
U
U
A
A
G
A
U
A
C
U
A
U
A
A
A
A
C
A
U
C
G
U
A
U
A
U
A
A
A
C
U
864nt
A
P5a
U
A
P5b
G
U
U
A
C
U
A
C
U
U
U
A
A
A
A
A
C
A
U
U U U U G U A U A A A
A
U
U
18nt
C
U
A
A G A A C G U A U U U
U
A
A
A
G
A
G
U
G
U
C
G
A
U
G
U
U
U
A
A
G
C
A
U
P5
U
C
G
C
U
A
A
A
U
A
G
U
G
A
U
A
A
A
U
A
A
A
A
A
A
G
G
C
A
A
U
G
U
A
A
U
U
U
A
A
U
A
P4
U
G
c
A
A
U
P3
C
C
U
g
C
G
P1
P2
P6
A
A
U
C
U
U
A
g
G
C
U
A
A
U
G
G
U U U
U
U U A U U A U U A C C U A G C U A U A
u
U
A
C C
A A G G A A A
5
’
-
G G
U
G G
U U C U U U U
G G A U C G
C C
U
A
U
A
A
U
U
G
U
U
U
U
59nt
C
A
A
A
U A A C C U C
G G
U U C A G G
27nt
-
3
’
a u u c c
A U U G G A G A A G A CA U A G U C C G A
U
A U A A A U U G
A
C C
A
A
U
A
U
A
P8
P7
U
A
U
A
P9
C
G
A
U
U
A
U
A
G
A
A
A
iAbi5

## Slide 6
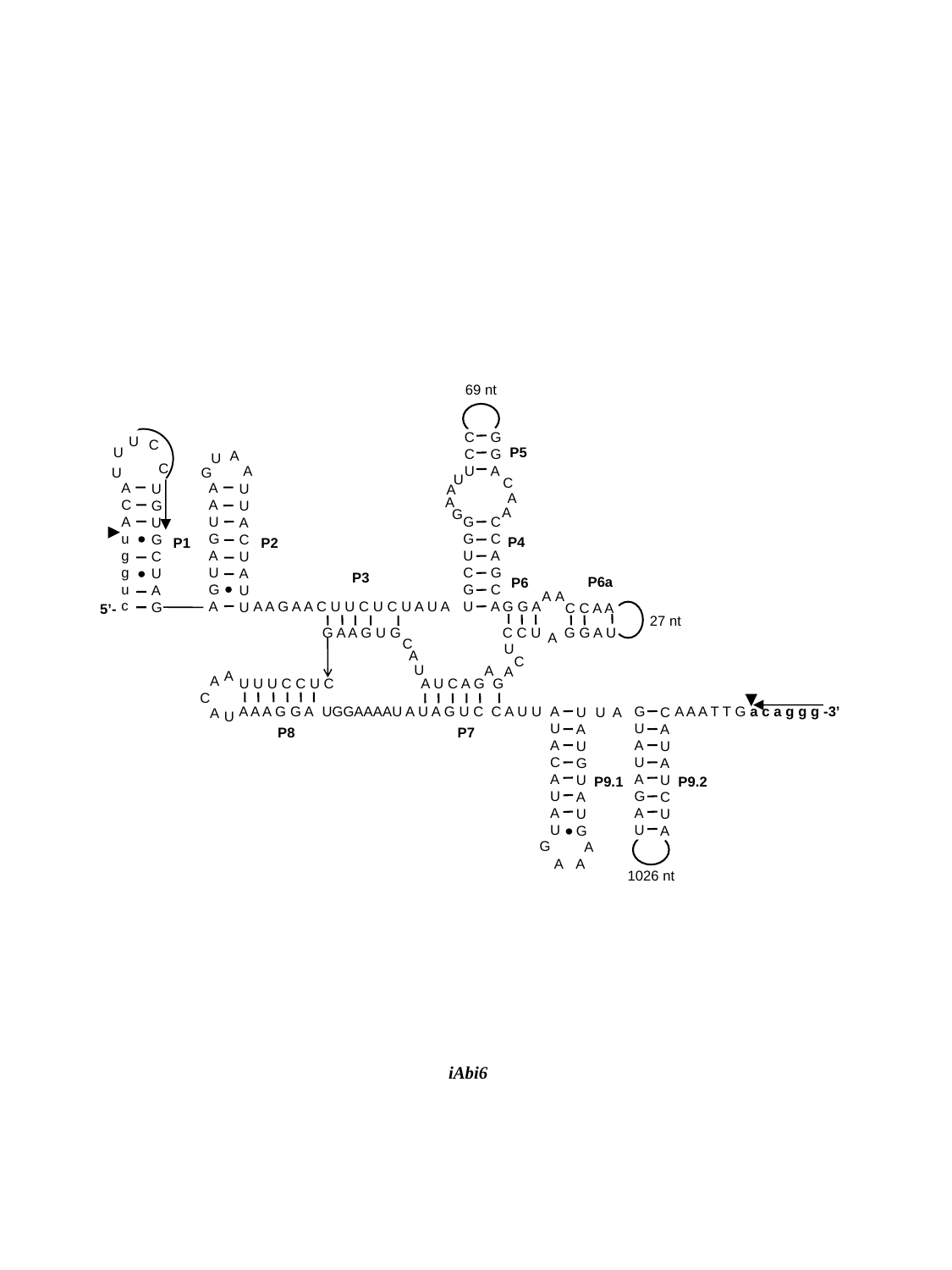

69 nt
U
C
P5
CCU
GGA
U
A
U
C
A
U
G
U
C
A
A
A
A
G
P4
P1
P2
UGUGCUAG
UUACUAUU
ACAugguc
AAUGAUGA
GGUCGU
CCAGCA
P3
P6a
P6
A
A
A A G A A C U U C U C U A U A
G G A
C C A A
5’-
27 nt
G G A U
G A A G U G
C C U
A A A T T G a c a g g g -3’
A
C
U
A
C
U
A
A
A
A U C A G G
A
U U U C C U C
C
UGGAAAAU A U A G U C C A U U
U A
A A A G G A
A
U
P7
P8
AUACAUAU
GUAUAGAU
UAUGUAUG
CAUAUCUA
P9.1
P9.2
G
A
1026 nt
A
A
iAbi6

## Slide 7
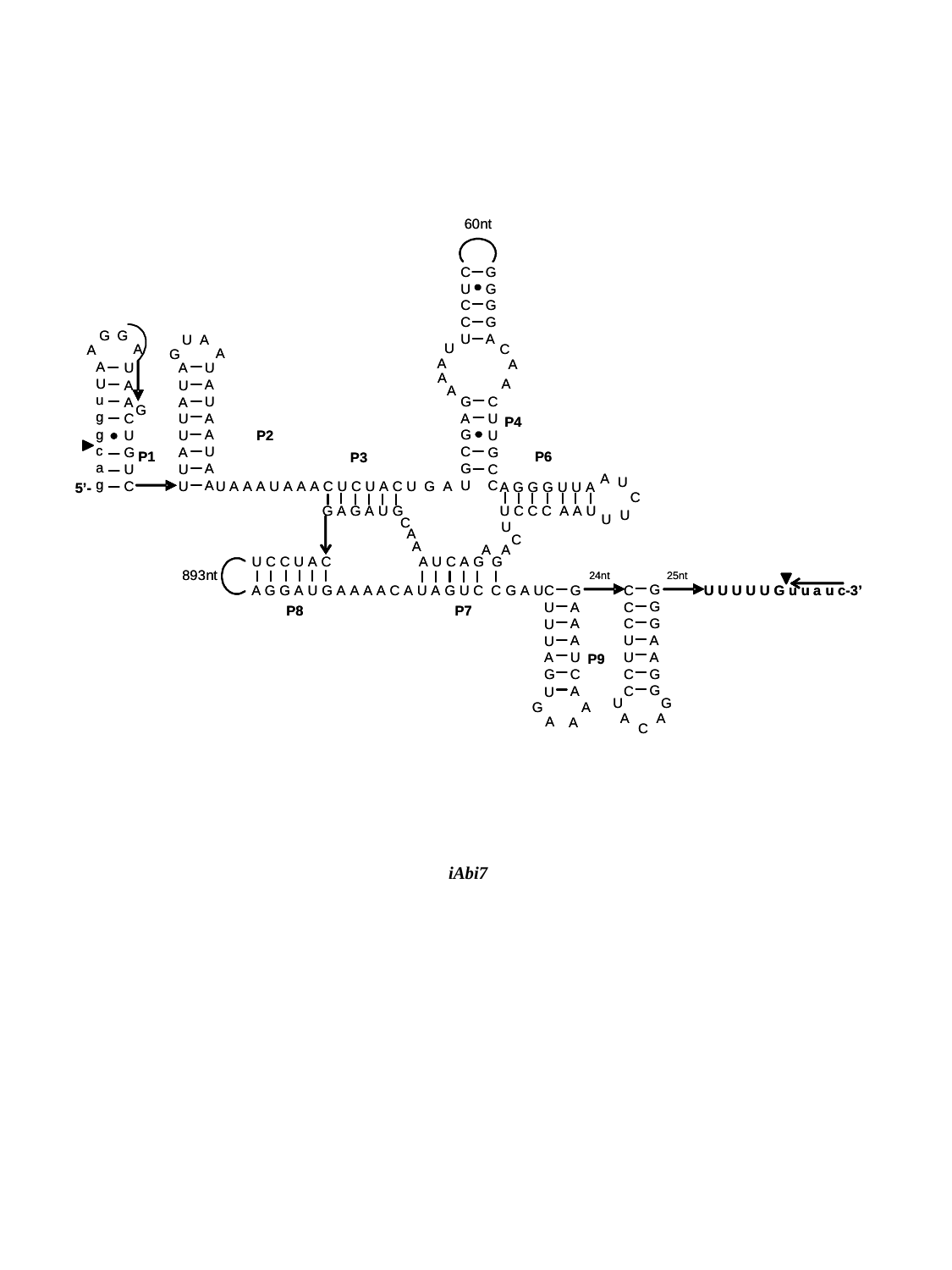

iAbi7

## Slide 8
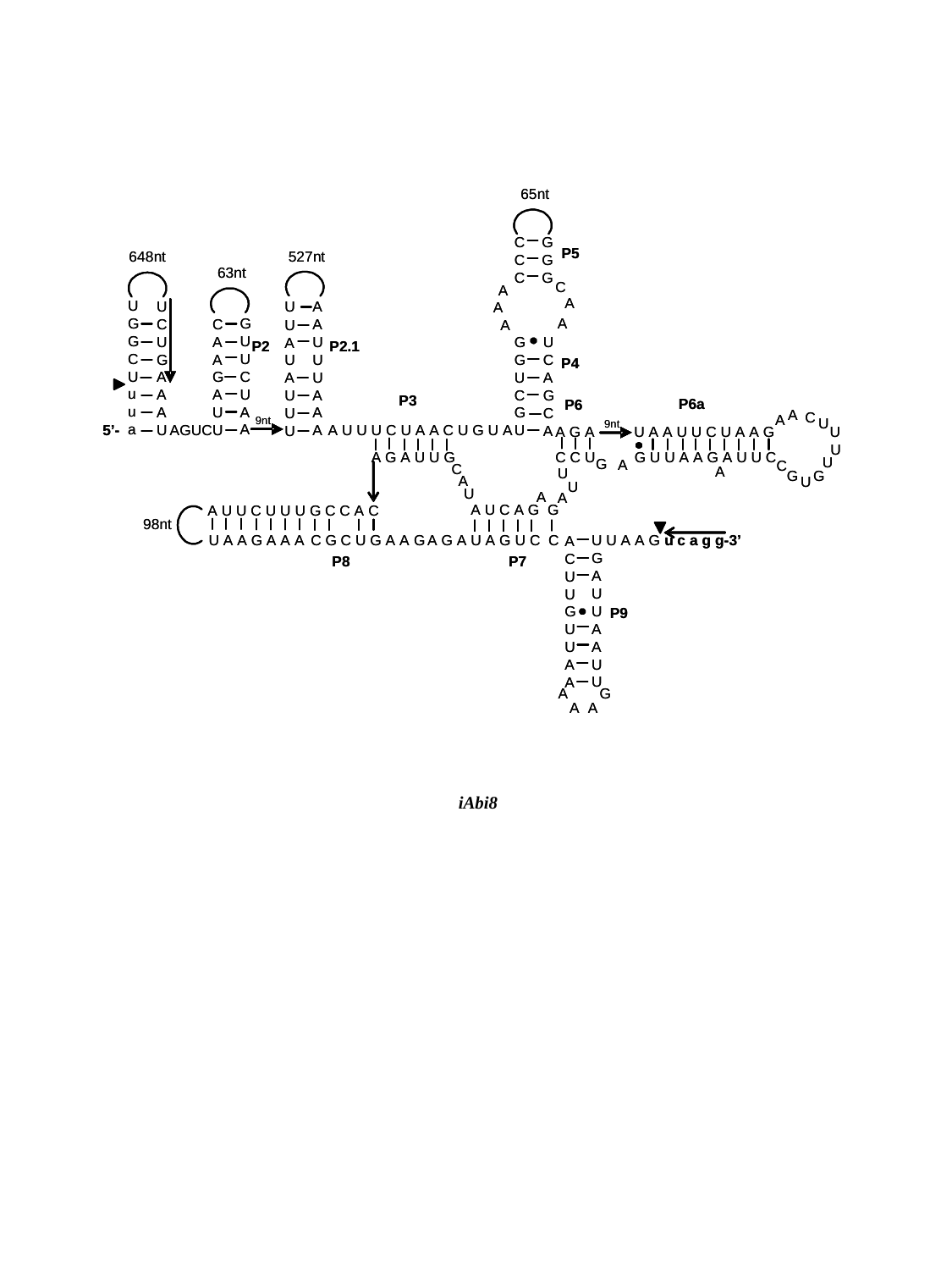

iAbi8

## Slide 9
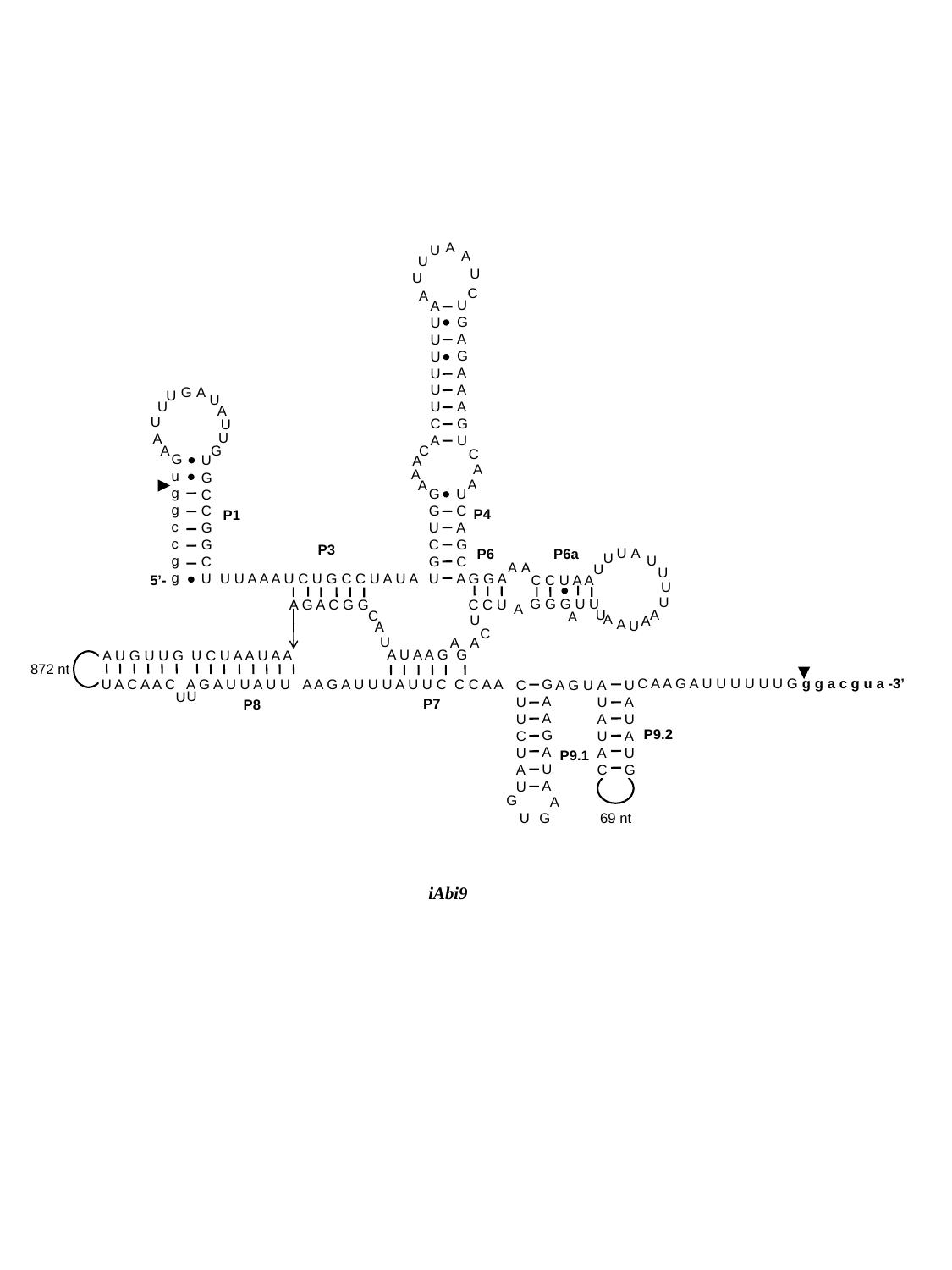

A
U
A
U
U
U
C
A
UGAGAAAGU
AUUUUUUCA
G
A
U
U
U
A
U
U
U
A
A
G
C
C
A
A
A
A
A
P4
P1
UGCCGGCU
Guggccgg
GGUCGU
UCAGCA
P3
C A A G A U U U U U U G g g a c g u a -3’
P6a
P6
A
U
U
U
A
A
U
U
U U A A A U C U G C C U A U A
G G A
C C U A A
5’-
U
G G G U U
A G A C G G
C C U
U
A
U
A
C
A
A
U
A
A
U
A
C
U
A
A
872 nt
A U A A G G
A U G U U G U C U A A U A A
A G U
A A G A U U U A U U C C C A A
U A C A A C A G A U U A U U
U
U
P7
P8
AUAUAC
UAUAUG
P9.2
GAAGAUA
CUUCUAU
P9.1
69 nt
G
A
U
G
iAbi9

## Slide 10
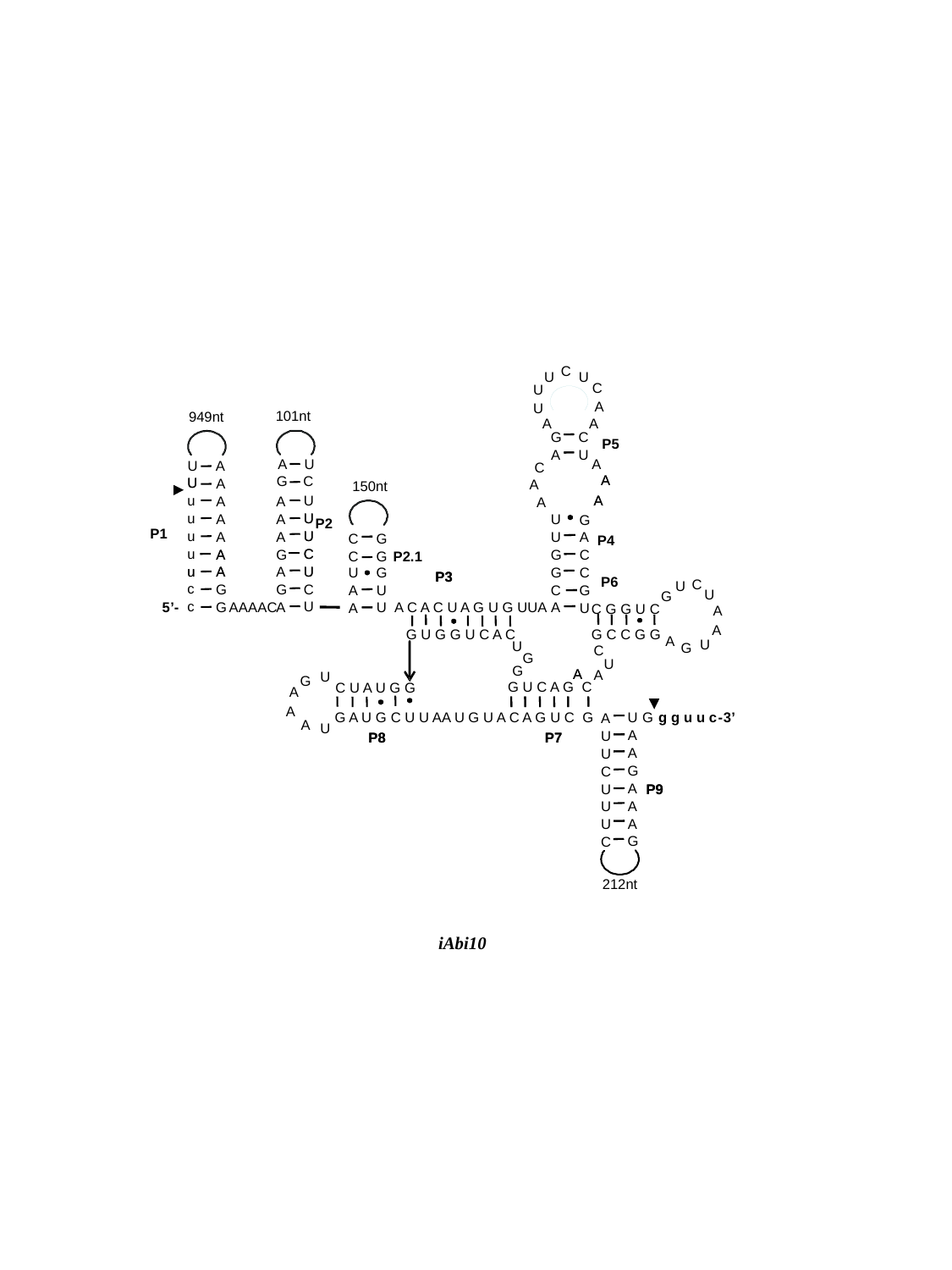

C
U
U
C
U
A
U
101nt
949nt
A
A
G
C
P5
A
U
A
U
A
U
A
C
A
A
G
C
U
U
A
A
150nt
A
A
u
U
A
A
A
u
U
U
A
A
U
G
P2
P1
u
U
U
A
A
U
A
G
C
P4
u
C
C
A
A
G
G
C
G
C
P2.1
u
u
U
U
A
A
A
G
G
C
U
P3
P3
P6
C
U
c
C
G
G
U
C
A
G
U
G
c
U
5
’
-
G
A
AAAAC
U
A C A C U A G U G UUA
A
A
U
C G G U C
A
A
G U G G U C A C
G C C G G
A
U
U
G
C
G
U
G
A
A
A
U
G
G U C A G C
C U A U G G
A
A
g g u u c
-
3
’
G
G A U G C U U AA U
G U A C A G U C G
U
A
A
U
A
U
P8
P8
P7
P7
A
U
G
C
A
U
P9
P9
A
U
A
U
G
C
212nt
iAbi10

## Slide 11
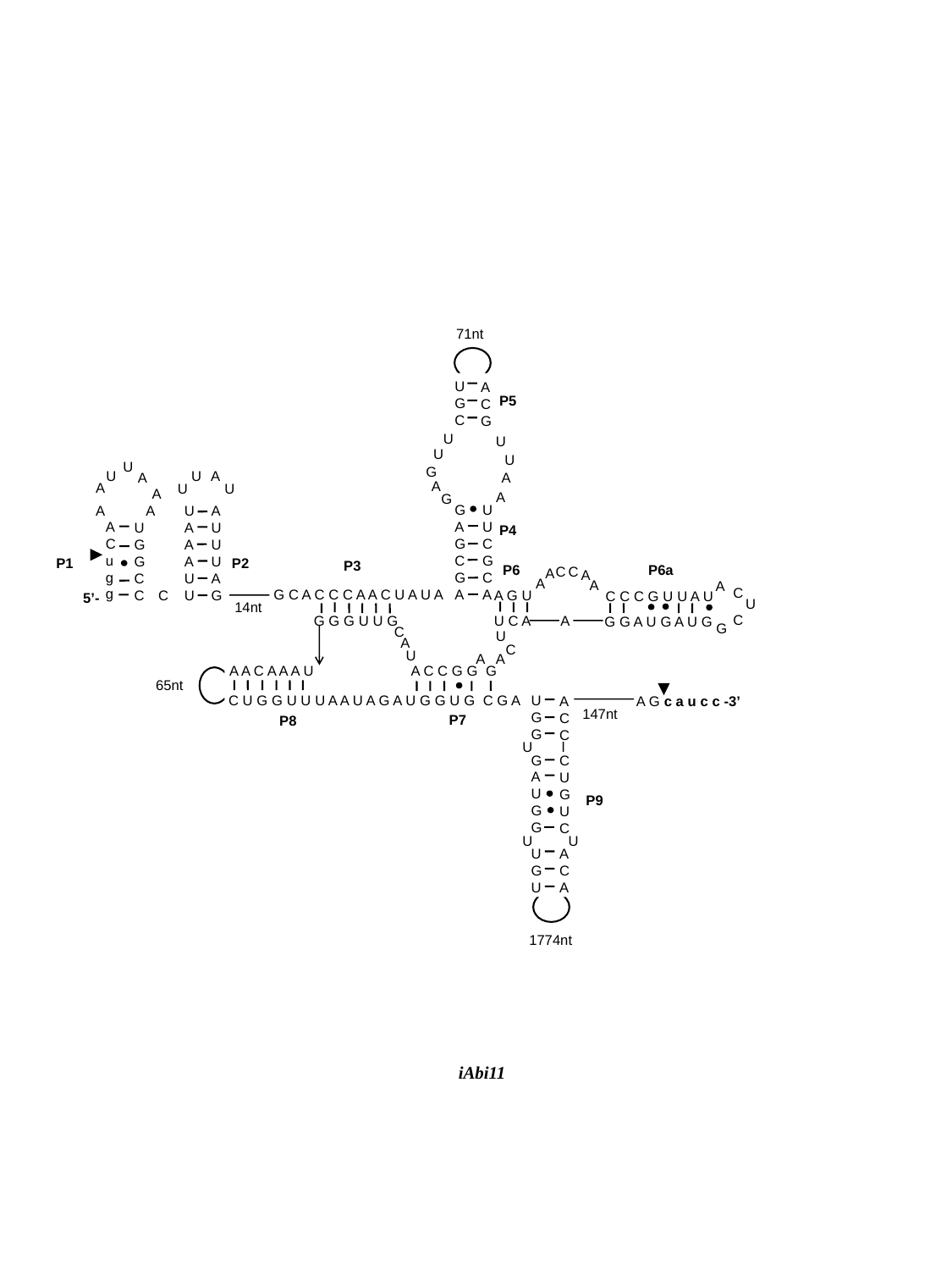

71nt
P5
UGC
ACG
U
U
U
U
G
U
A
U
U
A
A
A
A
U
U
A
A
G
A
A
P4
GAGCGA
UUCGCA
UAAAUU
AUUUAG
UGGCC
P2
P1
P3
ACugg
P6a
P6
C
C
A
A
A
A
A
G C A C C C A A C U A U A
A G U
C C C G U U A U
14nt
5’-
C
C
U
G G G U U G
U C A
G G A U G A U G
A G c a u c c -3’
C
A
G
C
U
A
C
U
A
A
A A C A A A U
A C C G G G
65nt
 C U G G U U U A A U A G A U G G U G C G A
147nt
P7
P8
U
UGG
GAUGG
UGU
ACC
CUGUC
ACA
P9
U
U
1774nt
iAbi11

## Slide 12
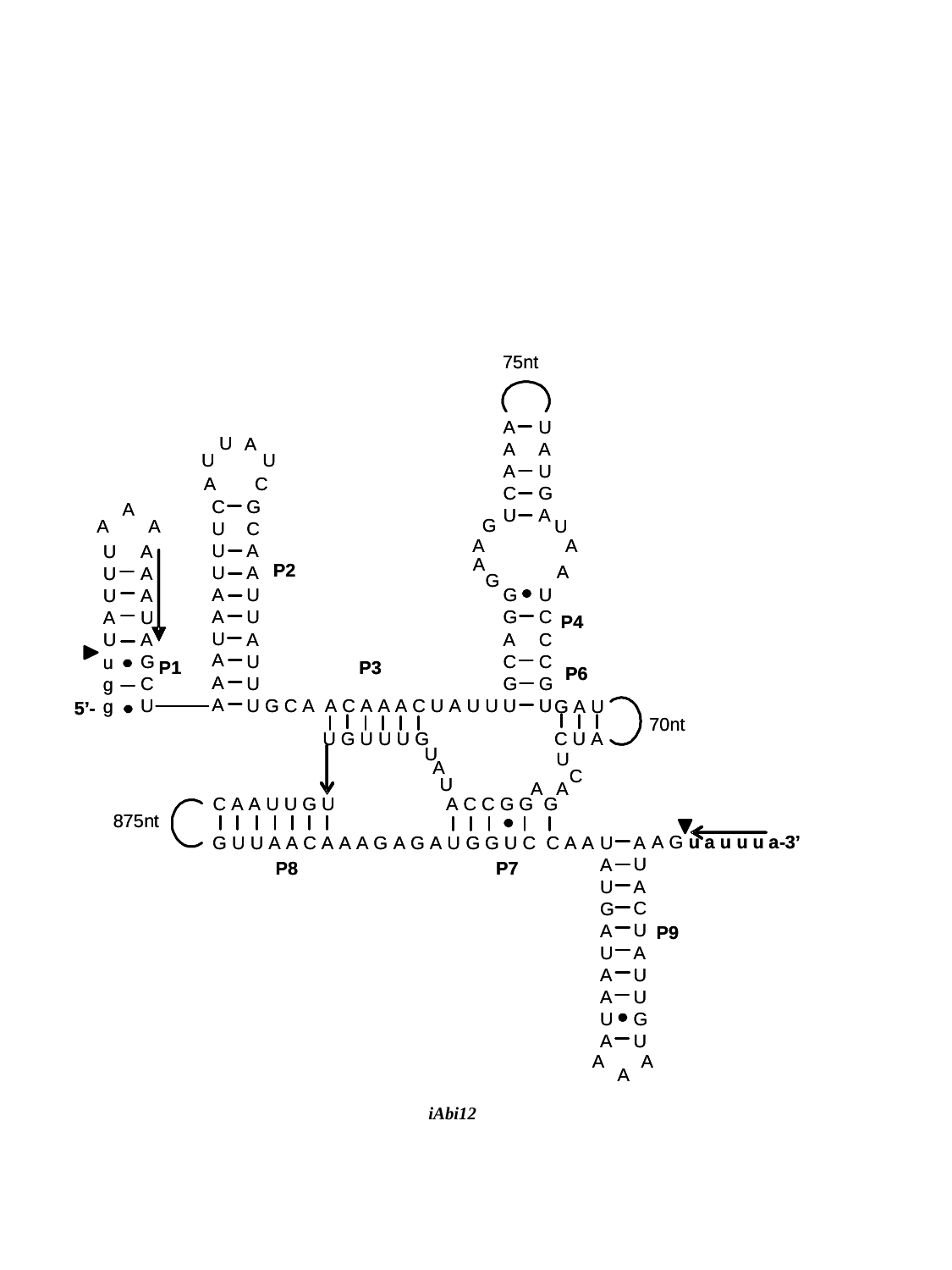

iAbi12

## Slide 13
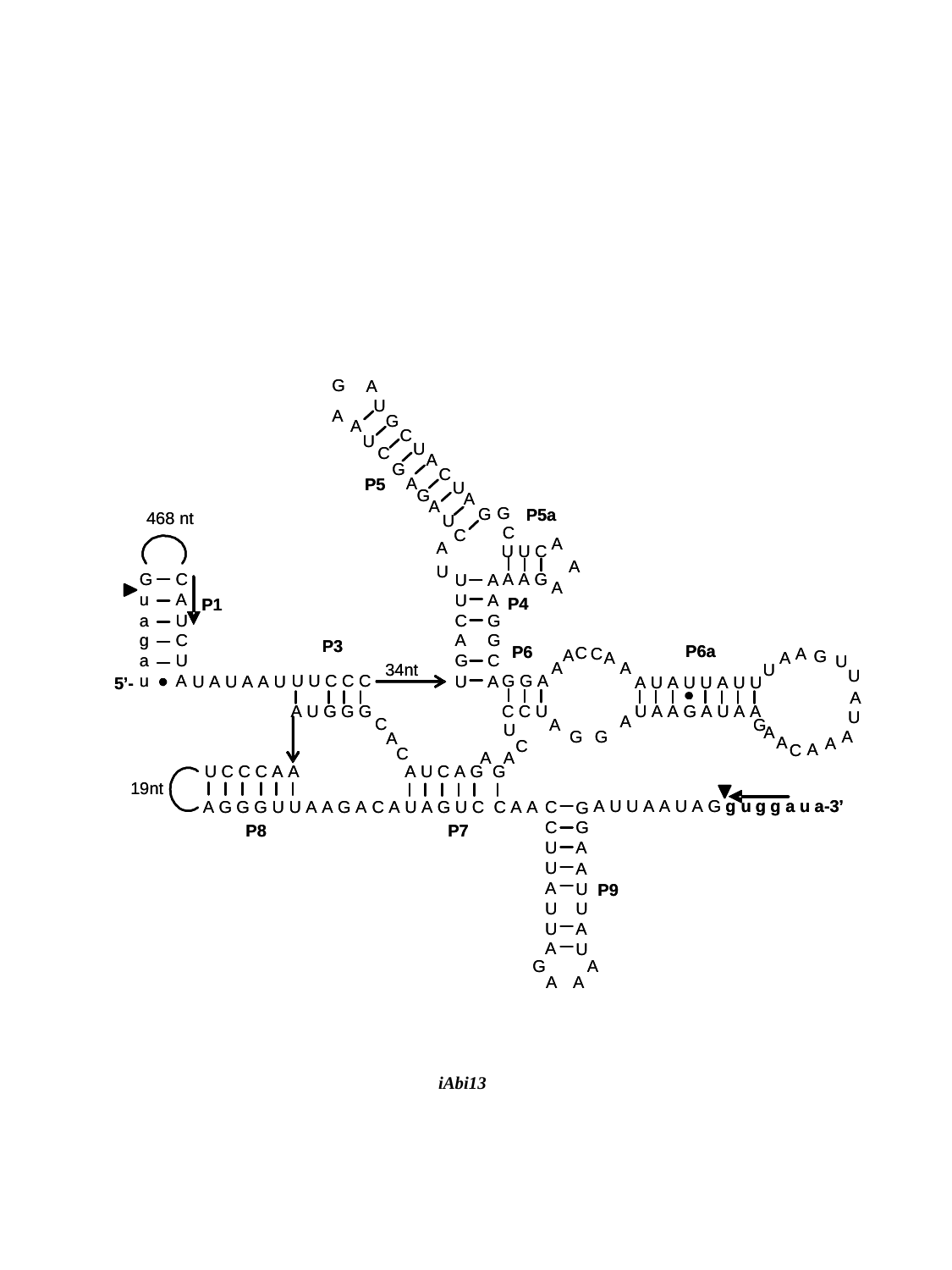

iAbi13

## Slide 14
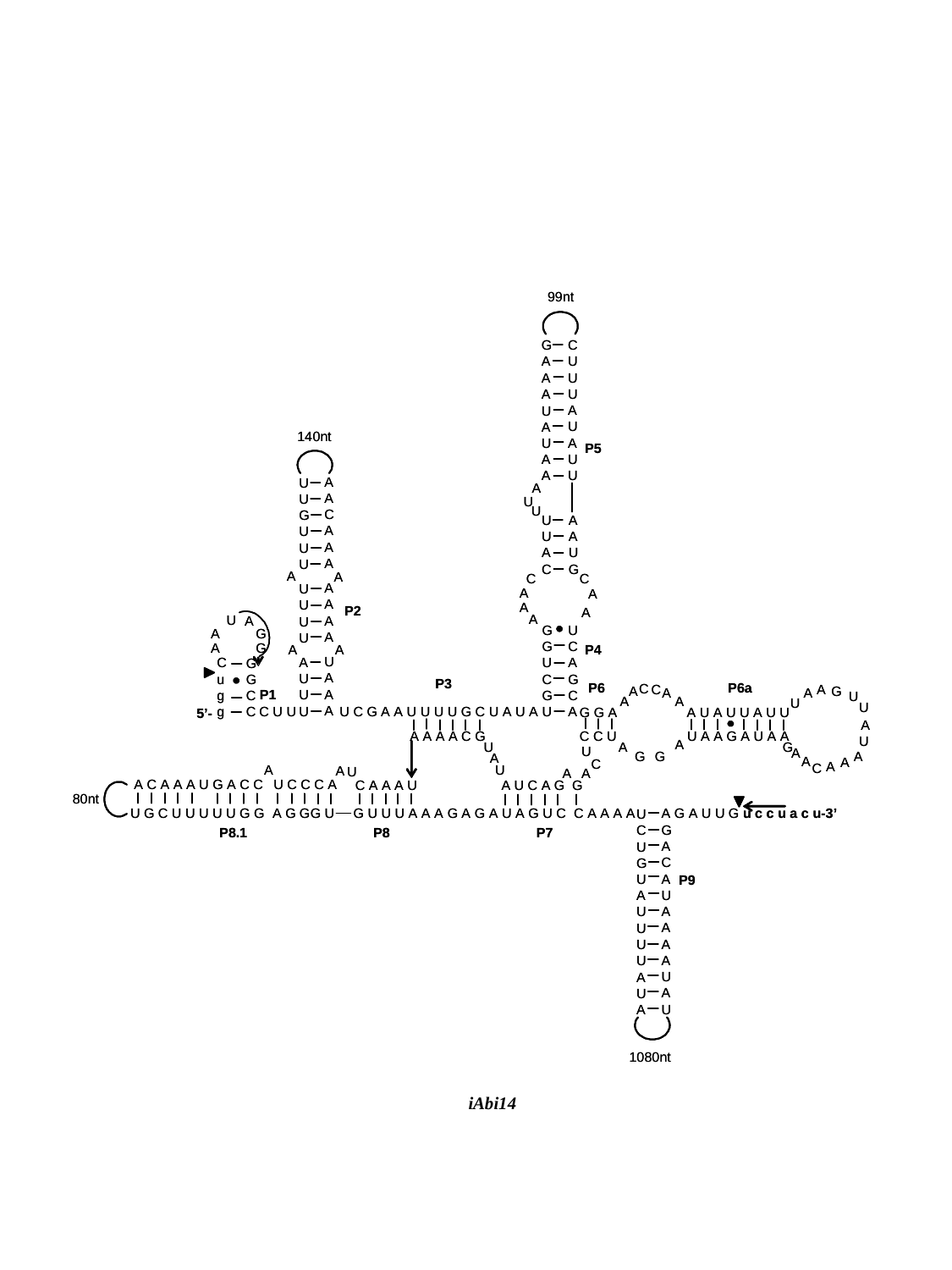

iAbi14

## Slide 15
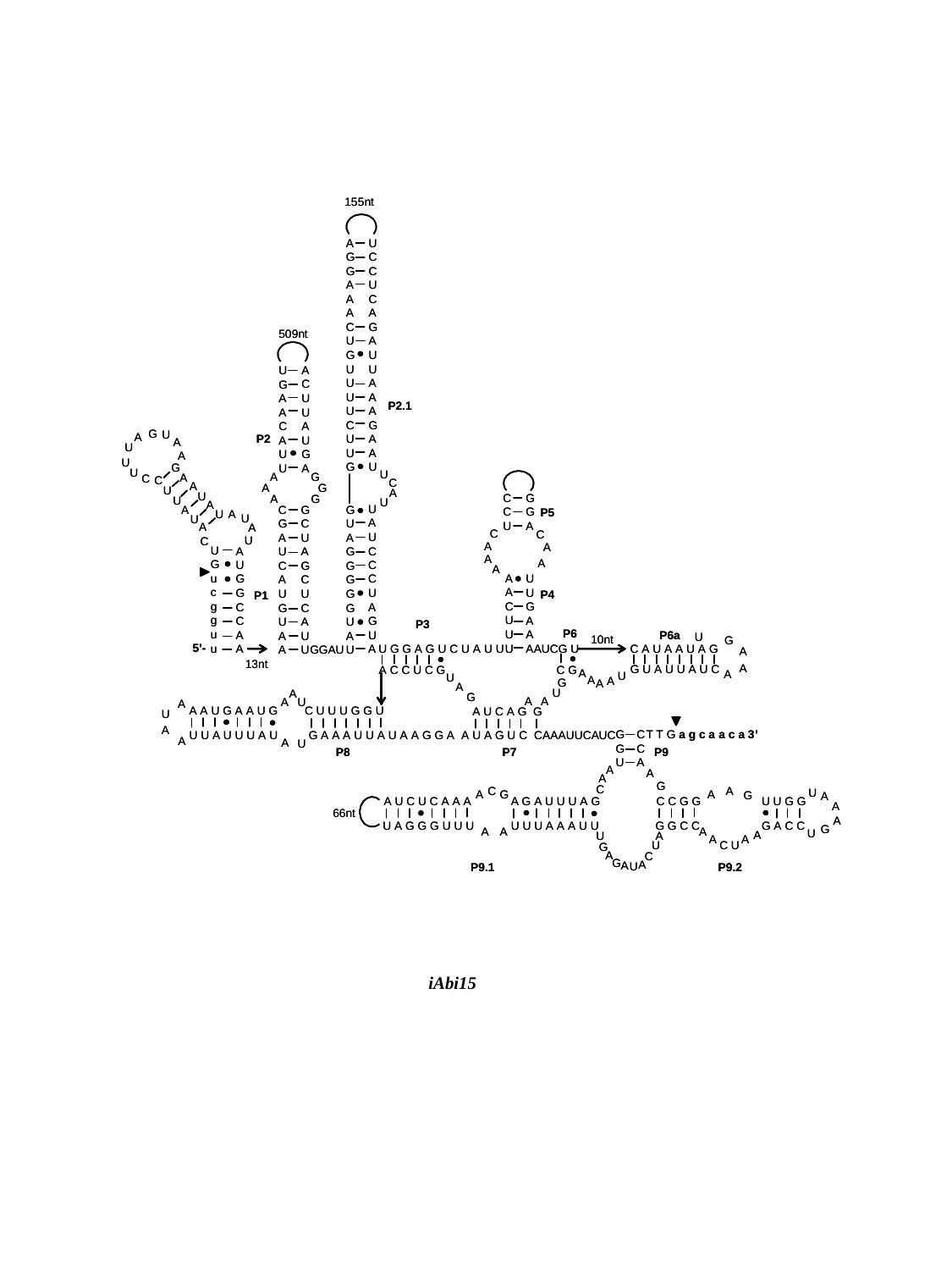

iAbi15

## Slide 16
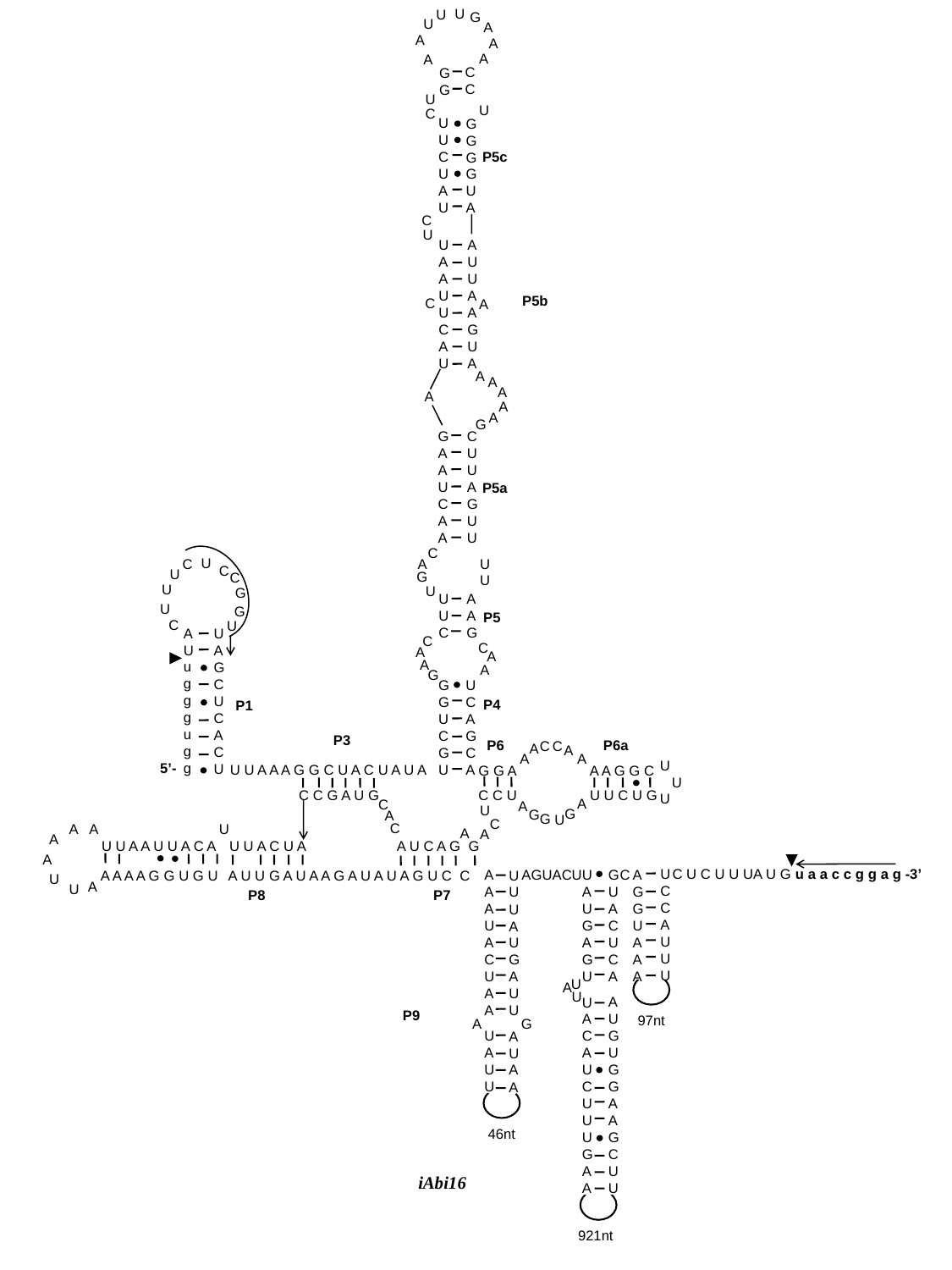

U
U
G
U
A
A
A
A
A
CC
GG
U
U
C
P5c
UUCUAU
GGGGUA
C
U
P5b
UAAUUCAU
AUUAAGUA
C
A
A
A
A
A
A
A
G
CUUAGUU
GAAUCAA
P5a
C
U
C
A
U
C
U
G
C
U
U
U
G
U
UUC
AAG
G
P5
C
U
C
C
A
A
A
A
G
UAGCUCACU
P4
P1
AUugggugg
GGUCGU
UCAGCA
P3
P6
P6a
C
C
A
C U C U U UA U G u a a c c g g a g -3’
A
A
A
5’-
U U A A A G G C U A C U A U A
G G A
A A G G C
U
U
U U C U G
C C G A U G
C C U
U
A
C
A
U
G
G
A
G
U
C
C
A
U
A
A
A
A
U U A A U U A C A
U U A C U A
A U C A G G
AGUACU
A
A U U G A U A A G A U A U A G U C C
A A A A G G U G U
C
U
A
U
P8
P7
UCCAUUU
AGGUAAA
AAAUACUAA
UAUU
UUUAUGAUU
AUAA
U
A
U
97nt
P9
G
A
GUACUCA
AUGUGGAAGCUU
UAUGAGU
UACAUCUUUGAA
46nt
921nt
iAbi16

## Slide 17
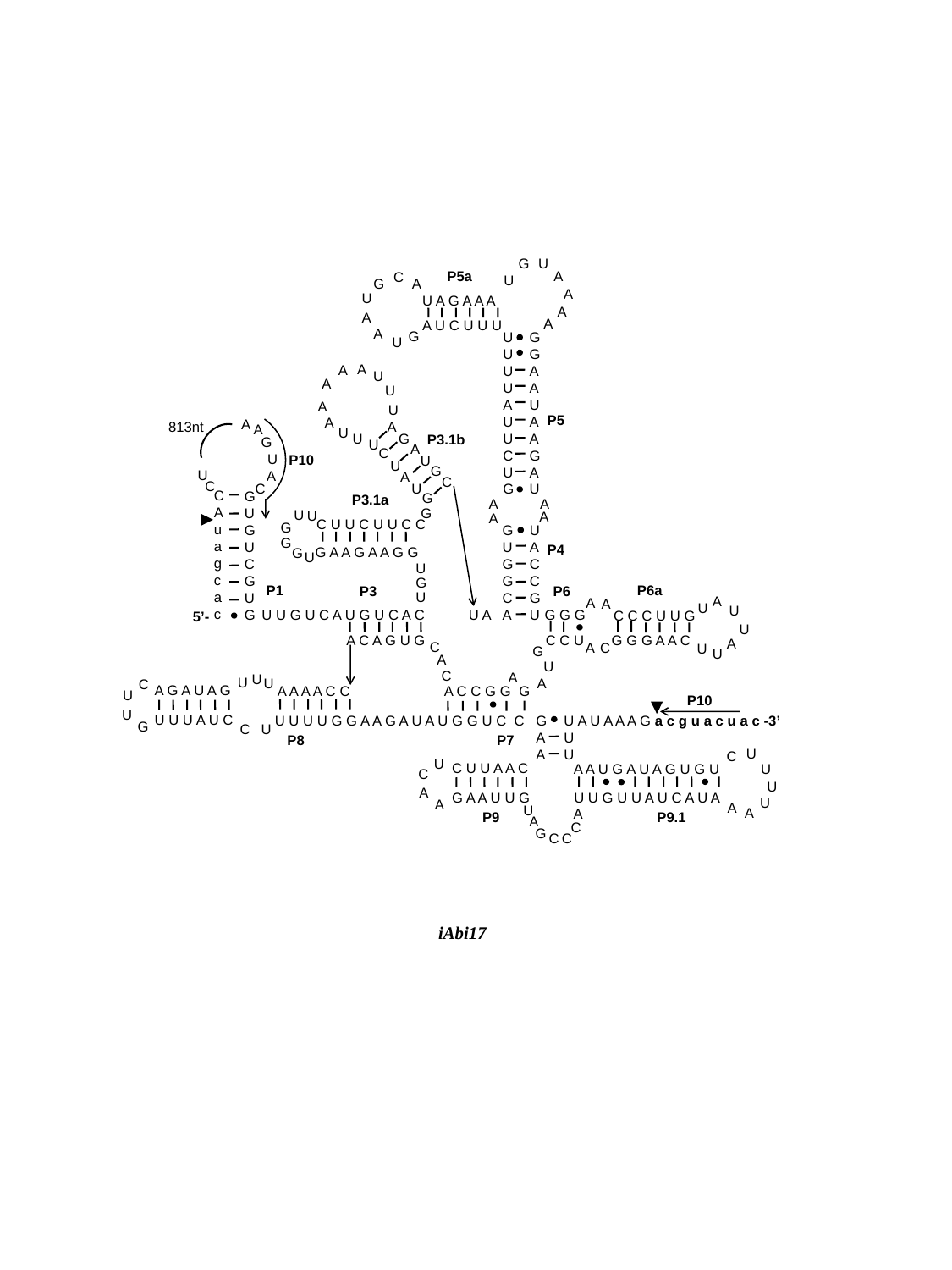

G
U
P5a
A
U
C
G
A
A
U A G A A A
U
A
A
A
A U C U U U
A
G
U
A
A
U
A
U
A
UUUUAUUCUG
GGAAUAAGAU
U
813nt
P5
A
A
A
A
U
P3.1b
G
U
G
U
A
C
P10
U
U
U
G
U
A
A
C
C
U
C
P3.1a
G
A
A
G
A
A
U
U
C U U C U U C C
G
G
P4
G A A G A A G G
GUGUCGUG
G
CAuagcac
U
GUGGCA
UACCGU
U
G
P6a
P1
P3
P6
U
A
A U A A A G a c g u a c u a c -3’
A
A
U
U U G U C A U G U C A C U A
G G G
C C C U U G
U
5’-
U
G G G A A C
C C U
A C A G U G
A
C
A
C
U
G
U
A
U
C
A
U
U
A
U
C
A G A U A G
A A A A C C
A C C G G G
U
P10
U
U U U A U C
U U U U G G A A G A U A U G G U C C
G
C
U
GAA
UUU
P7
P8
U
C
C U U A A C
U
A A U G A U A G U G U
U
C
U
A
G A A U U G
U U G U U A U C A U A
U
A
A
U
A
P9
P9.1
A
A
C
G
C
C
iAbi17

## Slide 18
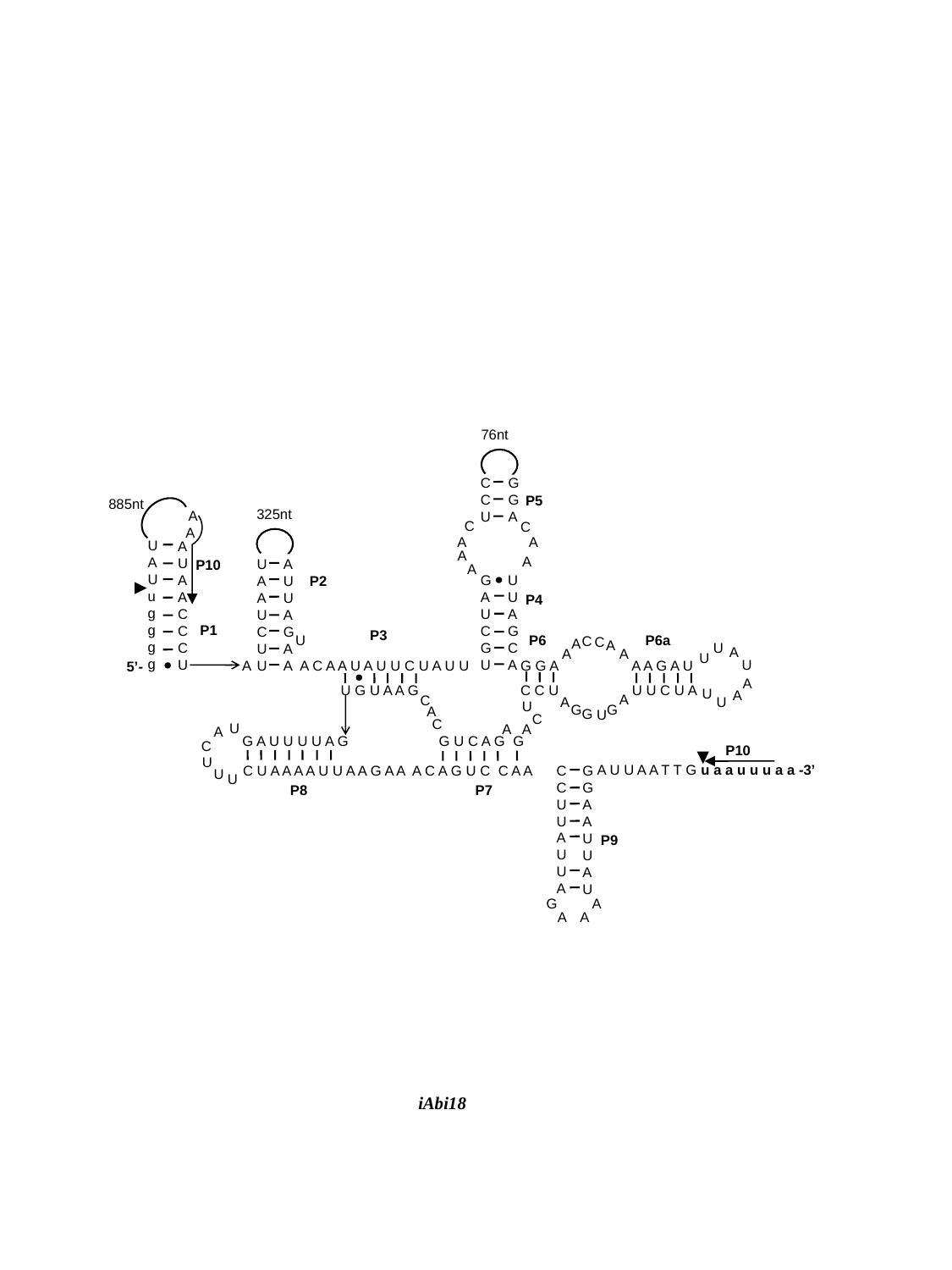

76nt
885nt
CCU
GGA
P5
325nt
A
C
C
A
A
A
A
A
P10
A
P2
P4
AUAACCCU
UAUugggg
UAAUCUU
AUUAGAA
GAUCGU
UUAGCA
P1
P3
P6
P6a
U
C
C
A
A
U
A
A U U A A T T G u a a u u u a a -3’
A
A
U
A C A A U A U U C U A U U
G G A
A A G A U
5’-
U
A
A
U U C U A
U G U A A G
C C U
U
A
A
C
A
U
U
G
G
A
G
U
C
C
U
A
A
A
G A U U U U A G
G U C A G G
C
P10
U
C U A A A A U U A A G A A A C A G U C C A A
U
U
P8
P7
CCUUAUUA
GGAAUUAU
P9
G
A
A
A
iAbi18

## Slide 19
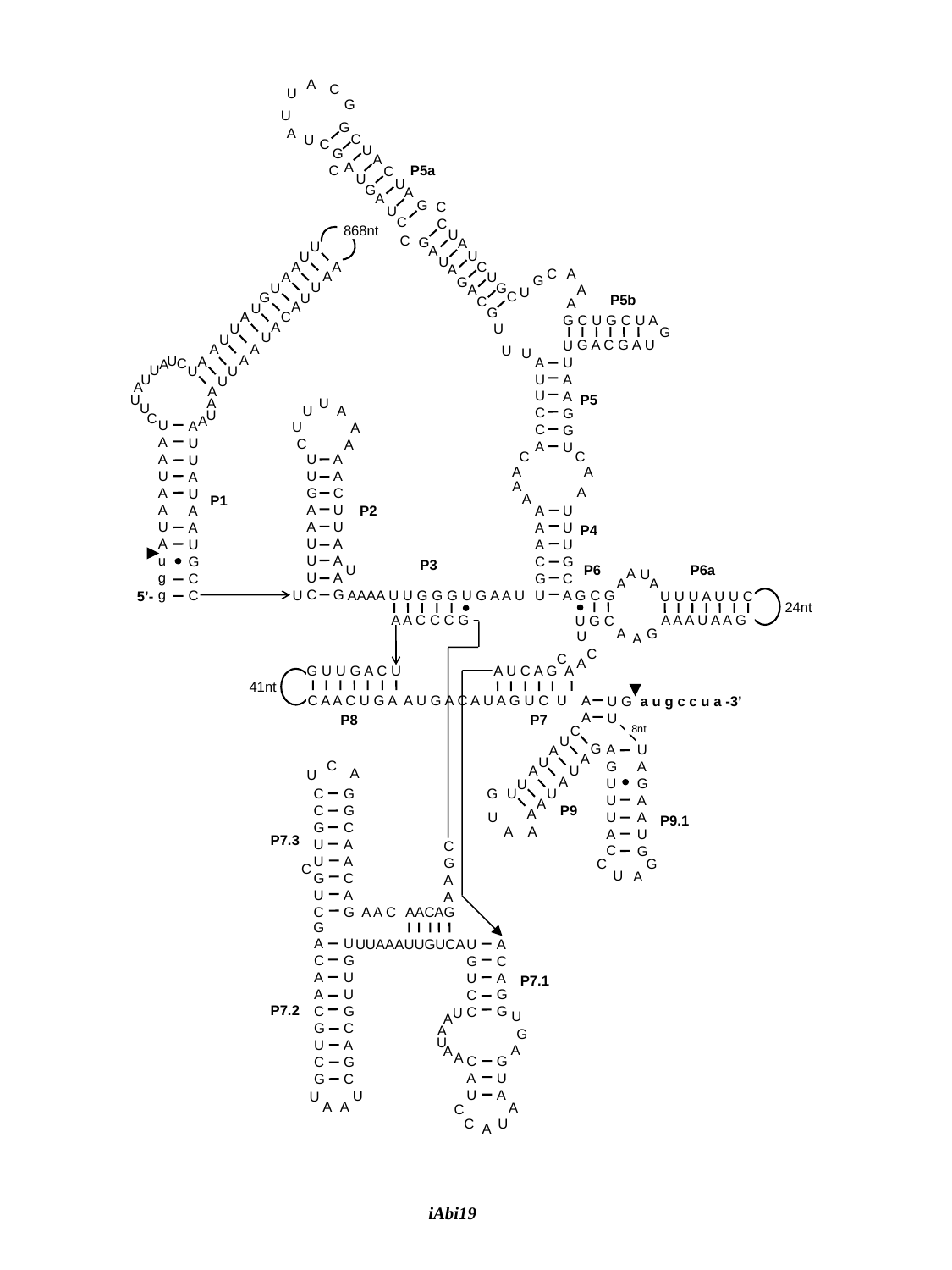

A
C
U
G
U
G
A
C
U
C
U
G
A
P5a
A
C
C
U
U
G
A
A
G
C
U
868nt
C
C
U
C
G
A
U
A
U
U
U
C
A
A
A
A
C
A
A
G
U
G
A
U
U
G
A
U
P5b
C
C
G
U
A
A
G
U
G C U G C U A
A
C
U
A
U
G
U
U
G A C G A U
U
A
U
A
A
U
A
A
C
U
U
U
U
U
A
UUAAGGU
A
P5
AUUCCA
U
A
U
U
U
A
U
C
A
U
A
C
A
C
C
A
A
A
A
A
P1
UAAUAAUAu g g
P2
AUUAUAAUGCC
UUGAAUUUC
AACUUAAAG
P4
AAACGU
UUUGCA
P3
P6
P6a
U
A
U
A
A
AAAA U U G G G U G A A U
G C G
U U U A U U C
5’-
24nt
U
G a u g c c u a -3’
A A C C C G
A A A U A A G
U G C
A
G
U
A
C
C
A
G U U G A C U
A U C A G A
41nt
C A A C U G A A U G A C A U A G U C U
AA
UU
P8
P7
8nt
C
U
G
A
A
U
C
U
A
A
U
A
U
U
G
U
AGUUUAC
UAGAAUG
A
P9
A
P9.1
U
A
A
P7.3
CCGUUGUC
GGCAACAG
G
C
CGAA
C
U
A
AACAG
 A A C
G
UUAAAUUGUCA
UGUCC
ACAGG
P7.1
P7.2
ACAACGUCG
UGUUGCAGC
U
U
A
A
G
U
A
A
A
GUA
CAU
U
U
A
A
A
C
C
U
A
iAbi19
