# Supplementary material for: The Agaricus bisporus cox1 Gene: The Longest Mitochondrial Gene and the Largest Reservoir of Mitochondrial Group I Introns
Source: PLoS One. 2010 Nov 18;5(11):e14048. doi: 10.1371/journal.pone.0014048 (PMC2987802; doi:10.1371/journal.pone.0014048)

**A**

I-AbiVII-P GQLARVNLLT L----QRLNV EHPNNIN--L TNSINTKVQG LRNNKELFYK WLVGFTDGDG TFSIAHQ--N G---KWSLAF KISQHEYNAR ILYFIKSQLG

I-AaeI-P RQFAWIIKLN FQIIHQRLHV EHLKKH---- -YSINVNKHT LSENKETFYQ WLVGFTDGDG TFSIVNQ--N N---KWSLTY KISQNTYNLR ILHFIKNQLK

I-HjeI-P RQYAWEQNVL MLNSHQRLNV EHPN-NIKIY DHSRSINRFK YNEDQDNFHQ WLVGFTDGDG SFSVIRSA-E G---KWTLFF KLTQSTYNLR AIYFIKKQLG

I-GzeII-P RQYAWELTKL MFRTHQRLNV EHPNENNNLF DHSKSINRFK YYENKDNFHQ WLVGFTDGDG SFSIVRVA-E R---KWTLFF KLTQSTYNLR AIHFIKNQLG

I-PosI-P ------RKKS LNIIHQRLNV GHLISHY--- -TTLTINKNS LSENKEIFHQ WLVGFTDGEG TFSIVRQ--N E---KWNLTF KISQSTYNLR VLNYIKKQLK

I-RorI-P GQSAWGKLDY STLSHQRLN- ---V------ ---------- MQPKNTEFQQ WLVGITDGDG SFSVLRQD-D ----KWTLTF KISQNTYNLR ALYYIKQQLG

I-PmaII-P RQCAWLENRR LFSSHQRLNG KHLY------ ---------- KDNNKIEFEQ WLVGFTDGDG NFHIAQQK-V GDTIKWNLGF KLTQSSYNAR VLYYIKKELG

I-MpeI-P GQSAWVSNNL SD----SSET TREVFSS--- --RIVNK--- -KNTNIEFEQ WLVGVTDGDG TFHFSEHL-P N---KWILNF KIGQSTYNLR LLYHIKSKLG

I-UmaII-P VVYNSVSFN- KN----PSET QRNVFSS--- --GRGVN--- -NSTN--FKE WLVGITDGDG TFYFAKTK-K G---IWSFSF QIGQSNYNIR LLYYIKSMLG

I-TinII-P GQSAWFGINT KNIFTKSSET TRSAFYSIFA QTTTPTQ--- -SHNLDQFYK WLVGVVDGDG TFHFSKTR-K G---VWTFYF KVRQSNYNLR LLYFIKKILR

I-SocI-P TRSAFNQTKL NN----NLNK YSNNFIS--- -KNSYIK--- -YYNKFDFEQ WLIGIIDANG YFYINK---E G---SCNFYF KIIINKYNLP LLYFIKSRLG

I-SpoI-P CFFLVYRTTY SFGVCLMKRF LFNKFFNRHP FTRVKSCFSS SSPSKFSFTQ WLVGFTDGDG CFSISKQKIK NGKNKWSLTF KLTQNLYNYR ILYFIKRNLG

I-RspV-P NMGIISQE-Y SNNSFKDLKL KNNKLNFK-- ----SSNFNK FDFSNDWFYE WLIGFTDGDG TFTIDRQK-- NG-TKWNLVY KVSQKSNNFK ILYFIKHKLS

I-CglII-P YASILYYNIY NNNMFQRLNV TKLIHLNN-- -IKI---NNI -KINNLNINE WLVGITDSNG IFNIYIN-NN N---KIIFTY KISLIINNIQ LLYKIKNYLK

I-KthII-P YASV----IF NYLTLQRLNV TKLKYYIK-- -KKYSLTNKT -NKSYFNINE WLVGITDGDG TFNIYTNMTH N---KIIFTY KISLNKKNTQ LLYKIKSYLG

I-CneeI-P LLNILILLSN NVYNYRIIRL YKLIFIHQ-- -RLNMIINII MNNKNLNFKS WLVGFTDGDG TFKI--NINK T---KVIYEY NITQNKNKEQ LLHKIKKELK

I-CgloII-P MRQSACE YLKNMSYQRL NVEPSFNFHE WLVGFTDGSG CFSITQQSQN -----LQCTF KITQSVYNYR VLYYIKKNIG

I-MpoIV-P NILTTEKSAQ SGRRPFQLRP KAKTFSTRGQ PAWGGAPHQR LNAEYLSFCR WLVGMTDGDG CFSIVLQNG- ----KYNLNF SIAQGKYNLR ILYYIKKILG

I-YliIII-P FKHIGQYACI LTNIHQRLHM AKQY------ --SNMSNSNN NPKDPFNFEE WLVGLVDGDG TFNVYVYPDK N---KGTFTF KISLSKYNAQ MLYYIKTKLG

I-MbrI-P MKNLINNEIK WAIGLIEADG YIGFYHNGNK ----KWIITL KVSLNSYNIK TIYKLKRIIG

I-PwiI-P MKNFKFYE WLGGLIDADG EFYISKS--- -----GYGSI EITMHIKEIQ TLYFIKSECQ

Consensus .......... .......l.. .......... .......... .......f.. WL!G.tDg#G .F.i...... .. .kw...f ki.q..y#.r .ly.IK..lg

I-AbiVII-P IGNINK-EDK ---TKMVNYR IRDRKKLLEI IFPIFDKNPL LTSKYFDYLK FKEAFNILED SNLTKTQKDE LMYNL----- ------VKKV PSESYISPAW

I-AaeI-P VGSIYI-EKG ---GNIGHFR IRDLKTLESV IFPIFDKYSL LTSKHFNYIK FKKAQQILSN SSLNKLDKDT LIYNL----- ------INSK PSVDYISPIW

I-HjeI-P VGSVYI-DSD ---CNKADFR IRDRKSIGSK IIPIFDKYPL LTSKYFSYQV FKKAYEILEN PNLSTKEKDN LLLNL----- ------KSEQ MPMDYISPAW

I-GzeII-P VGSIYV-DSD ---CNKADFR IRDRKTIGAT ILPIFDKYPL LTSKYFSYLK FKKAYEILEN PNLSTQDKDN LLLAL----- ------QKEQ MPLDYISPAW

I-PosI-P VGNINI-EKN ---KKICNFR IRDRSILFNI IFPIFDKYPL LTSKKFDYDN FKKAYFILSD SKLNSIEKDN FIFKL----- ------LNEL KPIEYISSAW

I-RorI-P VGSVSV-ESN ---KDMGSFR IRDRKQLANI IFPIFDRYPL LTTKYFNYAK FKSAYAILED KELTKSQKNA QIETL----- ------LLIQ PDESYISPAW

I-PmaII-P VGSITK-DGN ---KLQ--FF IRDRKNIESV LIPIFDKYPL LTTKYFDYMK LKKALYILND TEISITCKNL KIQKL----- ------KDSK PPVDYISPAW

I-MpeI-P VGKVSV--CA ---DGMAEYR LRDVKKIIQH IIPLFDKYPL LTSKHYNYDL FKQAAFILTD TSISTADKHI SLTKL----- ------KSKV RPDNYISPAW

I-UmaII-P IGSVSVTHAK ---DNTAHYR VRNIQHIIQY ILPIFDTYPL LTSKYFNYDL FKKAILIMND SSLSNQEKDE KISYL----- ------KSQS LPDNYISPAW

I-TinII-P VGSVSVPNSK ---DNMAEYR IRDIKVISRV ILPIFDKYTL LTSKQFNYTN FRKALHIYTD PLLSSREKDL QLSFL----- ------KTLT IPQNYKSTAW

I-SocI-P VGWIRLPNNN ---ISYCTYQ LKNPKHIIES ILPILEKYPL LSSKYFYYIK FKNALLIYND STLTLNEKKS LISYF----- ------EIIN LPVNFIAPIW

I-SpoI-P IGSLYK-ESS ---TNTVIYR LRRREHLKKI I-DIFDQFPL LTKKYWDYYL FKKAFLILED ANLNSFEKNS KLEEI----- ---R--IEKK SLKQY-SPV-

I-RspV-P YGNITK--SN ---DNNWCFR IRDKKVLNNV IFPIFDKFPL VTIKYYDYEI VKKAYLILTD ESLDKNTRNK LKEDL----- ---YNLLKKG PDNNYLSPVW

I-CglII-P IGSINY-TN- ----NIVIYL INNNNDLLNI ILPIFDKYKL LTSKRFNYLL FKKCLLINNN NNFNQLLKIN LINNIINN-- -----NNNNK YNNNYISDSW

I-KthII-P VGSVTF-DNK ---TNIASYL IRDKKYLLNI IIPIFDEYPL LTTKRYNYLK FKECLLISNN NLLTQNDKLL LINNIKNKTS SLDLYSDSLK KYNIYISDAW

I-CneeI-P VGKIIK-YN- ----NKVSYI IRDKRHIERV IFPIFDKYPL LSHKYYDYIK FKECLLINND LTLSQKDKLL KIKYI----- ------SINN MPKDYISPIW

I-CgloII-P YGSIT--QDG ---FKRVQYC IRDTKILKQI ILPIFEIYPL HTSKHIVYVL WKQALLYPEL RSVVQSRILS ASYLTC---- ---LDNLFEV KPIEVTKQSD

I-MpoIV-P VGSVNM---H ---KTIGVYR IRDRRKLAEI IFPIFDQYPC LTSKQFHYER FRQAHRILED PKLLTEHKYS ICEQL----- ------RSAT LPETYVNPVW

I-YliIII-P VGSVTITSNN SLDKNMITFR IRNTKHLKDI IFPIFDKYPL LTIKRYKYLI FKECVLLSLN STLTQEEKYR QIRQI----- ------YKNK QSEDYISDAW

I-MbrI-P YGSIHKS--- ---KNMVTWK ITNKETIKNH ILPILSQFNF RGLKYYQVMY LKDAINIMES SK-NTNEKHE ILSKL----- -----KLESK DCLWRVSPIV

I-PwiI-P -GKVTLRKG- ---VNVARWR LHKQEPLIET LTKLLGNIRI -AKRQIQYQK ICKIYDIDYK IPHLLTYDNA WFSGFFTGKG CISINKTNFM AVISVSQKEK

Consensus vGs!...... .n...yr ird.k.l... i.pifdkypl ltsK.f.Y.. fk.a..i..d ..l....k.. ....l..... .......... ....yisp.w

I-AbiVII-P EI-------- ---------- ---------- ---IHNQVNN INDANKVMSK SWLIGFTEAE GSFYLVNKSA N--RMVHGFE ITQ-KLDLIV LSAIACILGI

I-AaeI-P SI-------- ---------- ---------- ---VNNEVSN FDAASKVMSK AWLIGFTEAE GSFYLVQKTQ G--RLVHGFE ITK-KLDKIV LIAIKHILGI

I-HjeI-P EA-------- ---------- ---------- ---VNYEVNN TNDAKSIMSK YWLIGFTEAE GSFYLVSKTS T--RIVHAFE ITQ-KLDSIV LKAISLILGI

I-GzeII-P KT-------- ---------- ---------- ---VNYEVND TNKAKSVMSK YWLIGFTEAE GSFYLVNKAS T--RIVHAFE ITQ-KLDFIV LKAIAHILGI

I-PosI-P RI-------- -------PLD TVGAFRFSNF ESLVDNTISN FENASKVMSK PWLIGFTEAE GSFYLVAKSK D--RLVHAFE ITQ-KKDKIV LLAIKYILHI

I-RorI-P NK-------- ---------- ---------- ---ITLPIAD ANEAGKVISK SWLIGFVEAE GSFYLVSKDA S--RIVHEFS ITQ-KLDRVV LEGIRHILHI

I-PmaII-P NN-------- ---------- ---------- ---AKLPLTN VDSLNNVMTK SWLVGFIEAE GSFYLTNKDS N--RIVHGFG LTQ-KLDKVV LESIRILLHI

I-MpeI-P NV-------- ---------- ---------- ---VNNNVSC LAEAQTVMTK SWLVGFSEAE GSFYLVTKSA G--RIVHAFE ITQ-KLDKIV LDSIGYLLGI

I-UmaII-P NN-------- ---------- ---------- ---VNNQVTS ILDAMSVMTK SWLIGFTEAE GSFYIVKKGP L--RLVHAFE ISQ-KLDHIV LEAIALILDI

I-TinII-P ES-------- ---------- ---------- ---KSVTTMT LDEINLIMSK EWLIGFTEAE GSFYLLKKGP T--RMVHMFE ITQ-KLDKIV LEAIRVILPM

I-SocI-P NK-------- ---------- ---------- ---VNYRVNS KDEALKVISK SWLIGYLESE GNFSIMSKGS D--RKVHVFK IIK-KMDSII LRSIALILDI

I-SpoI-P N--------- ---------- ---------- ---------- --LEKYL-TK SWLIGFIEAE GSFYLLQKSP V--RIIHGFE ITQ-NYEQPL LAQISEFLFN

I-RspV-P N--------- ---------- ---------- ---------- --DSNYLLSK PWVIGFWEAE GSFYIVKKEE N--RLCHGFG ITQ-KFDKKI LFQLSKIFHT

I-CglII-P YNIYKN---- ----YKNINI NN-------I KIINNILENN LLIINKIITK SWLIGYIETK SSFKLIKKDN ---KIIHYFN INIIKLDYIV IYSIKLLLNI

I-KthII-P ESIYKKWNIK EDLLYLPMNL NDGVLIYPHK DIINNINSIL INDVNNIITK SWLIGFIEAK GSFFILKKSS NPLRCVHSFE LTQ-KLDYIV IYSIKLLLNI

I-CneeI-P NNIN------ ---------- ---------- --YNNIKS-- INDIKDKISK SWLTGFIEAK GRFIINKDRD ---RYIHIFS IS--SLDPII IYSIKYIFHI

I-CgloII-P SDVR------ ---------- ---------- ---RAILSKS LSQKRLEPTK SWIVGFVEAD GSFFLTKKGS EG-DIVHTFC ITQ-KHDYAL LEQLKKRFHI

I-MpoIV-P T--------- ---------- ---------- ---------- ---ANMAIDI DWLTGFIEAE GSFFLYNRDG KQ-RISIAFG LTQ-KLDRPL LEVIRRRLHI

I-YliIII-P KGLT------ ---------- ---------- ---IEDLNKD KTLVKNIMSK AWLAGFIDAE GCFTYVKKDS TRNYTLFCY- YSKIWSNSII WYKIFIKNKC

I-MbrI-P FPILN----- --------ND INNIIKYNLK KNNELIKLIP KNSLINIIDP WWLSGFIEGD GSFKINDK-- ----LQIVFE LGQ-KNNSFI IWLIHKYLNI

I-PwiI-P DILE------ ---------- ---------- ---NIQYIFK GNISFDISLK IWIWQCNNFD CEYLLTYFEK N--NILNPYK QAKIRGFKRF LFYKAQKYHL

Consensus ..... ... .......... .......... .........k sWliG%.#ae gsF.l..k.. ...r.vh.%. itq.kl#.i. l..i...l.i

I-AbiVII-P K-TTS----- -KKT---YHT VVTTNSRSIE NIIKYY-N-- --STMKGMKS FEFRVWARSY VK---HKGDF TKLNEIRNKI RSRKLGTTIF TNS

I-AaeI-P S-TNV----- -KVTKLGTFT VVTTNSRAIE NIINYF-K-- --NTMKGMKS IEYRIWSRAY VK---HKGNF LALEKTRNLM RNMRSNRYTL LDMNVNKMKE

I-HjeI-P N-FAK----- -K---TTYYT VVTTNSRAIS NIIDYY-S-- --KTMKGMKA VEFRIWARSF VK---HKGNY EKLSKIRDNI RIMRKVRLNE NFLVHKKDKD

I-GzeII-P S-VSR----- -KKLRASRHT VVTTNSRAIS NIIDYY-S-- --KTMKGIKA VEFRIWARSY VK---YKGNY EKLNSIRENI RVFRQIRLRS

I-PosI-P N-TKV----- -QHKKSG-YT IVTTNSRAIE NIIKYY-G-- --NTMKGIKS LEYRIWARAY IK---HKGNF MALNHIREKV RIMRKKYI

I-RorI-P S-TKVV---- -YKEKYNHYM IDTTNSRAIS NVSKYF-F-- --NTMKGMKG VEYRIWSRSF NK---HKGDY SQLVKIQKVL RGLRKVRANN TLWTSKSPKG

I-PmaII-P S-TKVI---- -YKEIYNHYI LDTTNSRAIE NIIEYF-N-- --NTMKGVKS LEYKIWARSY VK---YKNNY NNLYKIRNFV RKLRRNLEE

I-MpeI-P K-VIK----- ----KKSYFT VGTTNAKHIS NIILYF-H-- --KTMKGMKS LEYRIWARSF NKIKAGQARF EYLTKVRNQM RNIRSIRLDK NFQIINNSLL

I-UmaII-P K-VTT----- ----KKTYMT VVTTNYKSIE NIISYF-F-- --KTMKGMKA LEYRIWARSF NK--KGSGRF EYMTKIQNLM RNIRSIRLDK NFTKK

I-TinII-P K-VYI----- ----KRGYNT CVTTNHKSIG IIVEYF-K-- --DTMKGMKS LEYRIWARSY AK----DLTF IELTKVRDHM RAIRSIRNNK KSSEMKV

I-SocI-P K-FYN----- ----KQTYIM VTTQNKGSIL SLIHYL-K-- --NTMKGIKS LEYRIWSRSF LK---TKQDN IQLSKIQKLM NNIPSIRLDK NLKIKKVFKN

I-SpoI-P SQISPK---- -IKSKKNSLI TNYSLSTSSK ERMLFL-SSY FENCFKGVKS LEFKIWSRSL RK----NYNF EQLLRARDLI RKLKNKYSRG SQHPKDK

I-RspV-P S--SKV---- -RYNKKGFFS LDSTGHRANL NIVNYF-YDE KINYFIGIKS LQFTIWRRTL KF----RGQY IKLLRIRELL RALLN

I-CglII-P NINI------ -NN-----YK LELFDNKSIE YIINYLKFNN HKSRLLGIKS YEYLIWKRSY YK---YKNNN KKLLIIKILL DKNI

I-KthII-P NSSIVIKSPS ISNKSNLFYK LETTNNSNIE YIINYFRYKN YKSVFLGIKS FEYRIWSRTY IK---HKGNY IKLLQIKNLL QKHRKNY

I-CneeI-P NAKV------ --KHKEIFNM IETANSRNIN NIIRYFIKSN NKNIFYGIKS YEFNLWKKSY LN---YRNNN SKLCKIQQSI NRFI

I-CgloII-P KAKIK----- -HNQKTKAYV LETTNTRNIR FLIQFF---- -HGTFKGMKS LEYRIWARSF VK---NKGLS DELLTVQNQL RKLKKVKV

I-MpoIV-P PTQII----- ---ERPHFYR LETTHSRAVL GISQLF---- -QGRFKGMKS LEVEALVESR LL

I-YliIII-P KCNM------ ----KQKRFL FIKYN

I-MbrI-P QSKIK----- -VKKDNSYTT LSTKDPKIIN KLIILL---- -KGKLLGIKS FEYSIWSRAF R-----TNKM NKLCKAKEIL NKIRKQRNSI K

I-PwiI-P DPL------- --KRKRLLHF IKKFNSIYSL

Consensus ...... ...k..... ..ttns..i. .ii.yf.... .....kg.ks .ey.iw.rs. .k ..... ..l..i.... r......... ..........

**C**

| HE  HE | I-AbiVII-P |
| --- | --- |
| I-AaeI-P | 57%a/87%^b^ |
| I-PosI-P | 56%/84% |
| I-HjeI-P | 55%/83% |
| I-UmaII-P | 50%/81% |
| I-RorI-P | 52%/81% |
| I-GzeII-P | 55%/81% |
| I-MpeI-P | 48%/80% |
| I-KthII-P | 47%/80% |
| I-PmaII-P | 48%/77% |
| I-TinII-P | 44%/75% |
| I-RspV-P | 41%/75% |
| I-SocI-P | 39%/73% |
| I-CgloII-P | 38%/73% |
| I-SpoI-P | 41%/72% |
| I-CneeI-P | 35%/71% |
| I-YliIII-P | 32%/68% |
| I-MbrI-P | 26%/66% |
| I-CglII-P | 35%/65% |
| I-MpoIV-P | 37%/63% |
| I-PwiI-P | 22%/63% |

**B**


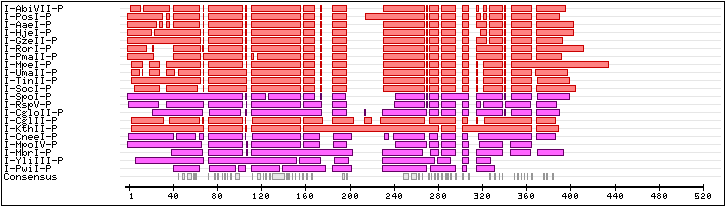

Supplement: Figure S3 — Alignement (A) and representation of the Total Score (B) obtained when comparing the 21 intact HE of the most widely distributed Pcl K. The Total Scores higher than 200 are in red, those between 80 and 200 in pink. In C are reported the percentages of aa identity (a) and aa similarity (b) between I-AbiVII-P and the 20 others orthologous HE. (0.07 MB DOC) [file pone.0014048.s003.doc]
